# Recognising, quantifying and accounting for classification uncertainty in type 2 diabetes subtypes

**DOI:** 10.1007/s00125-025-06486-4

**Published:** 2025-07-25

**Authors:** Tim Mori, Oana P. Zaharia, Klaus Straßburger, John M. Dennis, Knut Mai, Stefan Kabisch, Stefan Bornstein, Julia Szendroedi, Matthias Blüher, Svenja Meyhöfer, Jochen Seissler, Andreas Birkenfeld, Norbert Stefan, Michael Roden, Robert Wagner, Oliver Kuß, and the GDS Group, Hadi Al-Hasani, Bengt Frederik Belgardt, Gidon J. Bönhof, Gerd Geerling, Christian Herder, Andrea Icks, Karin Jandeleit-Dahm, Jorg Kotzka, Eckhard Lammert, Wolfgang Rathmann, Sabrina Schlesinger, Vera Schrauwen-Hinderling, Sandra Trenkamp

**Affiliations:** 1 Institute for Biometrics and Epidemiology, German Diabetes Center, Leibniz Center for Diabetes Research at Heinrich Heine University Düsseldorf, Düsseldorf, Germany; 2 German Center for Diabetes Research (DZD), München-Neuherberg, Germany; 3 Institute for Clinical Diabetology, German Diabetes Center, Leibniz Center for Diabetes Research at Heinrich Heine University, Düsseldorf, Germany; 4 Division of Endocrinology and Diabetology, Medical Faculty and University Hospital Düsseldorf, Heinrich Heine University Düsseldorf, Düsseldorf, Germany; 5 Institute of Clinical & Biomedical Sciences, University of Exeter Medical School, Exeter, UK; 6 Department of Endocrinology and Metabolism, Charité – Universitätsmedizin Berlin, corporate member of Freie Universität Berlin, Humboldt-Universität zu Berlin, Berlin, Germany; 7 Berlin Institute of Health, Berlin, Germany; 8 German Institute of Human Nutrition Potsdam-Rehbrücke, Nuthetal, Germany; 9 Department of Internal Medicine III, Carl Gustav Carus University Hospital Dresden, Technical University Dresden, Dresden, Germany; 10 Department of Medicine I and Clinical Chemistry, University Hospital of Heidelberg, Heidelberg, Germany; 11 Helmholtz Institute for Metabolic, Obesity and Vascular Research (HI-MAG) of the Helmholtz Zentrum München, University of Leipzig and University Hospital Leipzig, Leipzig, Germany; 12 Department of Medicine I, University Hospital Schleswig-Holstein Campus Lübeck, Lübeck, Germany; 13 Diabetes Center, Medical Clinic and Policlinic IV, University Hospital, LMU Munich, Munich, Germany; 14 Department of Internal Medicine IV, University Hospital Tübingen, Tübingen, Germany; 15 Centre for Health and Society, Faculty of Medicine, Heinrich Heine University Düsseldorf, Düsseldorf, Germany

**Keywords:** Classification uncertainty, Clusters, German Diabetes Study, Precision medicine, Relative entropy, Subtypes, Type 2 diabetes mellitus

## Abstract

**Aims/hypothesis:**

Despite continued interest in precision diagnostics and type 2 diabetes subtypes, the challenge of uncertainty in the classification of individuals into subtypes remains. This study introduces a novel method for quantifying and accounting for classification uncertainty in type 2 diabetes subtypes.

**Methods:**

Building on recommendations from the ADA/EASD Precision Medicine in Diabetes Initiative, we quantified classification uncertainty using the normalised relative entropy (NRE), computed from distances to cluster centroids. A lower NRE value indicates greater uncertainty in an individual’s cluster assignment. We examined the NRE in a cohort of 859 individuals with recent-onset type 2 diabetes from the prospective, observational German Diabetes Study (GDS) and compared it across previously identified diabetes subtypes, defined by age, BMI, HbA_1c_, HOMA-IR and HOMA-B. Predicted 10 year CVD risk (SCORE2-Diabetes) of the subtypes was evaluated with and without accounting for classification uncertainty.

**Results:**

Individuals with mild age-related diabetes (*n*=395) and mild obesity-related diabetes (*n*=316) had a median NRE of 0.155 (95% CI 0.142, 0.177) and 0.119 (95% CI 0.107, 0.131), respectively. By contrast, individuals with severe insulin-resistant diabetes (*n*=130) and severe insulin-deficient diabetes (*n*=18) had a lower median NRE of 0.086 (95% CI 0.075, 0.108) and 0.082 (95% CI 0.071, 0.109), respectively. After weighting individuals by classification certainty, the proportion of variation in SCORE2-Diabetes explained by the subtypes (*R*^2^) increased from 17.4% (95% CI 12.8, 23.0) to 31.5% (95% CI 26.4, 37.1). The predicted 10 year CVD risk of the mild age-related diabetes subtype increased from 10.3% (95% CI 9.8, 10.7) to 11.6% (95% CI 11.2, 12.0).

**Conclusions/interpretation:**

The NRE provides a means to quantify and compare individual classification uncertainty in type 2 diabetes subtypes. Classification uncertainty varied between subtypes and individuals with type 2 diabetes, and accounting for it improved the ability of the subtypes to predict 10 year CVD risk.

**Graphical Abstract:**

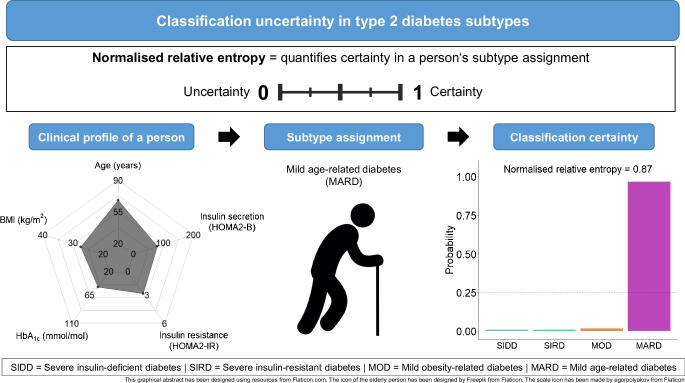

**Supplementary Information:**

The online version contains peer-reviewed but unedited supplementary material available at 10.1007/s00125-025-06486-4.


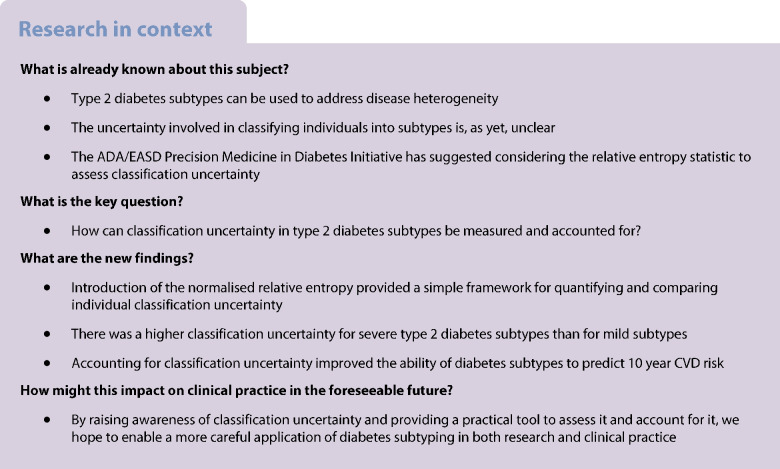


## Introduction

The original concept of subtyping adult-onset diabetes [1] has been validated with gold-standard measurements [2] and replicated in more than 20 studies of ethnically diverse populations [3, 4]. These subtypes (or ‘clusters’) were initially derived from the Swedish All New Diabetics in Scania (ANDIS) cohort using *k*-means clustering and comprise severe autoimmune diabetes (SAID), severe insulin-deficient diabetes (SIDD), severe insulin-resistant diabetes (SIRD), mild obesity-related diabetes (MOD) and mild age-related diabetes (MARD) [1]. The latter four subtypes (SIDD, SIRD, MOD and MARD) correspond to non-autoimmune diabetes subtypes and provide a subclassification for type 2 diabetes, which is the focus of the present study [3]. Individuals are assigned to the type 2 diabetes clusters based on five continuous clinical features using the ‘nearest centroid approach’ [1, 2]. This corresponds to a hard clustering approach (i.e. each individual is exclusively assigned to a single cluster). Such hard clustering approaches have a number of limitations [5–9]. As dimensionality reduction approaches, they inherently waste information compared with directly considering the clinical features on their original, continuous scale [10]. On the one hand, such a simplification is desirable, since a discrete cluster membership is easy to communicate and the different subtypes might be associated with different pathophysiological processes [11]. On the other hand, there may still be considerable heterogeneity between individuals within a cluster [12].

**Fig. 1:**
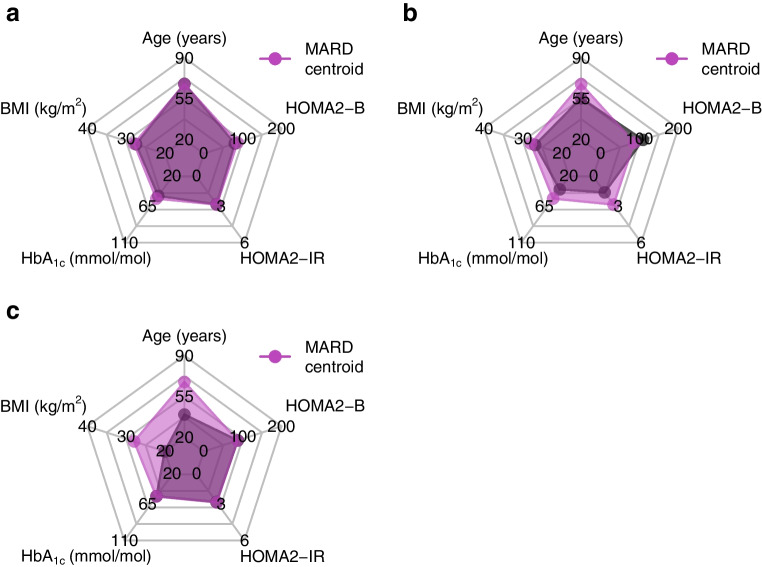
Spider charts of three individuals from the GDS classified as having MARD. The charts display their clinical profiles (dark purple/black) in comparison with the typical MARD profile from the nearest centroid algorithm (light purple). The individual in (**a**) closely aligns with the typical MARD profile, whereas the individual in (**b**) shows some deviations from it. The individual in (**c**) was assigned to the MARD subtype despite a younger age (40 years)

Beyond these limitations, hard clustering also introduces the challenge of classification uncertainty, which is often overlooked [13]. While the nearest centroid algorithm forcibly assigns any individual with type 2 diabetes into exactly one of the four clusters, individuals will differ in terms of how well they match with their assigned subtype [6]. This is illustrated in Fig. 1, which shows the clinical profiles of three individuals from the German Diabetes Study (GDS) classified as MARD [2, 14]. Accordingly, and as explicitly suggested by the ADA/EASD Precision Medicine in Diabetes Initiative (PMDI), discrete cluster assignments should be accompanied by a measure of classification uncertainty [13].

To date, only a few studies have attempted to quantify the classification uncertainty in type 2 diabetes subtypes [15–17]. Given the lack of practical tools available, we aimed to do the following: (1) introduce a novel, statistically robust method for quantifying classification uncertainty in aetiological type 2 diabetes subtypes; (2) apply it in the prospective, observational GDS cohort and compare classification uncertainty across subtypes; and (3) demonstrate how to account for classification uncertainty when comparing clinical outcomes between subtypes. In particular, we follow the proposal from the PMDI [13] to quantify classification uncertainty using the relative entropy statistic.

## Methods

### Quantifying classification uncertainty using the normalised relative entropy

**Fig. 2:**
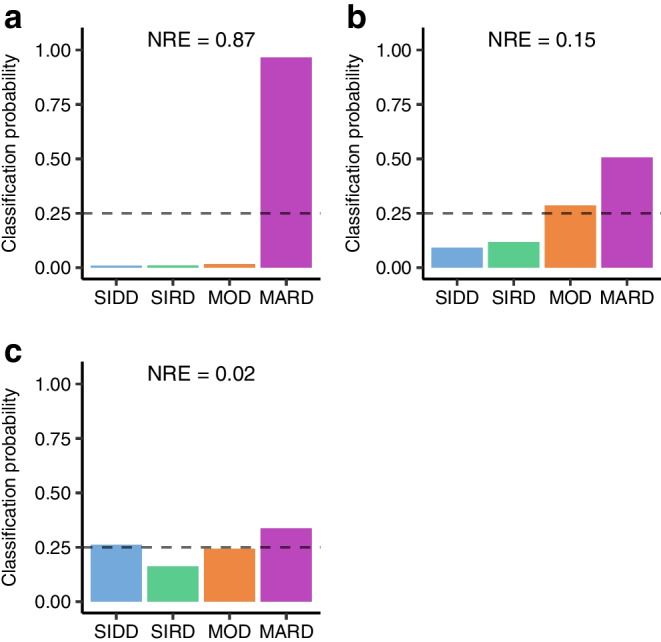
Classification probabilities and NRE for three example individuals from Fig. 1 who were assigned to the MARD subtype by the nearest centroid algorithm. The NRE quantifies the classification uncertainty on a scale from 0 to 1, with higher values indicating greater certainty. The black dashed line indicates the classification probabilities in a reference setting with complete uncertainty regarding an individual’s cluster assignment. The individual in (**a**) has high classification probability of 97% for the MARD subtype, whereas the individual in (**b**) has a somewhat lower classification probability of 51%. The individual in (**c**) has a low classification probability of 34% for the MARD subtype

The relative entropy is a statistical measure that can be used to combine the classification probabilities for the type 2 diabetes subtypes into a single number, to provide an overall measure of classification uncertainty [13]. To obtain an individual’s classification probabilities, we used information from the nearest centroid approach. First, the Euclidean distances were inverted to obtain a measure of individual similarity (rather than dissimilarity) to each of the four subtypes [18]. Then, these four similarity measures were normalised such that they sum up to one, thereby representing classification probabilities [18, 19]. For mathematical details, see the electronic supplementary material (ESM) Methods section titled ‘Classification probabilities based on the nearest centroid approach’. Figure 2 shows the resulting classification probabilities for the previously shown individuals from the GDS cohort (Fig. 1).

The relative entropy compares an individual’s classification probabilities with a hypothetical reference scenario representing complete classification uncertainty. In this reference scenario, the classification probability for each subtype is exactly 25% (black dashed line in Fig. 2), indicating that we have no information about which cluster an individual belongs to. The relative entropy then indicates how much more certain an individual’s cluster assignment is compared with this baseline of complete uncertainty. After normalisation [19], the relative entropy takes on a value between 0 and 1. A normalised relative entropy (NRE) of 0 indicates complete uncertainty in the individual’s cluster assignment, with all classification probabilities being 25% [20]. An NRE of 1 indicates complete certainty, with a 100% probability for the assigned cluster and 0% for all other clusters [19]. For an NRE between 0 and 1, a higher value indicates a greater classification certainty.

Conveniently, the NRE in our case can be computed using a basic calculator. For example, based on their classification probabilities, the NRE of the individual with MARD in Fig. 2c is computed as follows:

$$NRE\;=\;1\;+\;\frac{0.26\;\times\;\log_e\left(0.26\right)\;+\;0.16\;\times\;\log_e\left(0.16\right)\;+\;0.24\;\times\;\log_e\left(0.24\right)\;+\;0.34\;\times\;\log_e\left(0.34\right)}{\log_e\left(4\right)}=\;0.02$$

For other individuals, the NRE can be obtained analogously by plugging their classification probabilities into the numerator of the equation. It is important to note that the NRE is a conservative measure of classification uncertainty, as it requires high classification probabilities to achieve a high NRE. For example, the classification probabilities of the individual with MARD with a moderate fit (51% probability for MARD) translate into an NRE of only 0.15 (Fig. 2b). Similarly, even the individual with a good fit (97% probability for MARD) achieves an NRE of just 0.87 (Fig. 2a), a value further from the maximum NRE than one might intuitively expect. Additional information on this behaviour is available in the ESM Methods section titled ‘Relationship between the normalised relative entropy and classification probabilities’. For guidance on the interpretation of different NRE values, we provide a reference chart in ESM Fig. 1. Additional practical examples based on GDS individuals from the other three subtypes (SIDD, SIRD, MOD) are available in the ESM Figs 2–7. The general mathematical formula as well as further methodological details are provided in the ESM Methods section titled ‘Quantifying classification uncertainty using the normalised relative entropy’.

### Study population

We examined the classification uncertainty of individuals with recent-onset type 2 diabetes in the GDS cohort [14], in which the diabetes subtypes have been extensively studied [2, 21–26]. The GDS is a multicentric, prospective observational study of individuals with newly diagnosed diabetes in Germany [14]. Participants are recruited from multiple sites across the country, including Düsseldorf, Tübingen, Berlin, Potsdam, Munich, Heidelberg, Dresden, Leipzig and Lübeck. The GDS investigates subphenotypes of diabetes and risk factors for diabetes-related comorbidities using comprehensive demographic, clinical and metabolic assessments. For this study, we used data from the baseline visit, at which participants were 18–69 years old with a known diabetes duration of less than 12 months. Type 2 diabetes was diagnosed according to ADA recommendations. At baseline, the GDS excludes individuals with HbA_1c_ >75 mmol/mol (>9%) or acute heart, liver, renal or other severe diseases. More detailed information on the GDS is available in the published cohort profile [14].

For the current analysis, we excluded individuals who had incomplete information on the variables needed for cluster assignment [1]: GAD autoantibody measurements; sex (self-reported); age at diagnosis; BMI; HbA_1c_; fasting plasma glucose; and fasting C-peptide. Fasting plasma glucose and fasting C-peptide were used to calculate HOMA-IR and HOMA-B indices [27]. Individuals with plasma glucose and C-peptide values outside the acceptable range for HOMA calculations (glucose 3.0–25.0 mmol/l, C-peptide 0.2–3.5 nmol/l) were excluded. Moreover, we excluded individuals with GAD antibodies (cut-off 2 U/ml) [28], as those are automatically classified as SAID in the diabetes subtype framework [2]. Details on the procedures and measurements in the GDS cohort have been reported previously [2, 14]. Besides the variables needed for cluster assignment, we also extracted further clinical information necessary to predict the 10 year CVD risk based on the SCORE2-Diabetes model [29]: smoking status; systolic BP; total cholesterol; HDL-cholesterol; and eGFR.

The GDS received approval from the ethics committee of the Faculty of Medicine at Heinrich Heine University Düsseldorf (reference no. 4508), along with approval from other study locations. The study was conducted in compliance with the Declaration of Helsinki, registered on ClinicalTrials.gov (registration no. NCT01055093), and all participants provided written informed consent.

### Statistical analysis

Participants were assigned to the type 2 diabetes clusters using the nearest centroid approach [1, 2] and classification uncertainty was quantified with NRE as described above. In the nearest centroid approach, sex-specific centroid information was used for subtype assignment as described previously [1]. We visualised the distribution of NRE in the GDS cohort using a boxplot and compared it with the classification probability for the assigned cluster. Additionally, we plotted the NRE values across the type 2 diabetes subtypes and compared them using median values with 95% CIs. To examine potential drivers of low classification certainty, we plotted the NRE against the continuous clinical features used for cluster assignment (age, BMI, HbA_1c_, HOMA-IR and HOMA-B) separately for each subtype.

Using SCORE2-Diabetes as an example, we illustrated how to account for classification uncertainty when comparing clinical outcomes between type 2 diabetes subtypes. Specifically, we fitted a weighted linear regression model to predict SCORE2-Diabetes based on the diabetes subtypes, with each person being weighted by their NRE value (weighted least squares) [30]. This ensured that individuals with higher classification certainty contributed more to the analysis, while those with lower certainty contributed less. We then compared the results with a standard linear regression model, in which we did not weight by classification uncertainty and each individual contributed equally (ordinary least squares). Then, we compared the *R*^2^ values of the two models to assess the proportion of variation in SCORE2-Diabetes explained by the subtypes. The two *R*^2^ values served as a measure of predictive ability of the subtypes with and without accounting for classification uncertainty. Additionally, we compared the predicted SCORE2-Diabetes values from the two models across the subtypes. All analyses were performed using R version 4.3.1 [31] and SCORE2-Diabetes was calculated using the RiskScorescvd R package version 0.2.0 [32].

## Results

Of 1143 participants with type 2 diabetes, 258 were excluded due to incomplete data on clustering variables, seven due to fasting plasma glucose and C-peptide values outside the acceptable range for HOMA calculation and 19 due to GAD antibodies. The final study cohort included 859 participants, the majority of whom were assigned to the MARD (*n*=395) and MOD (*n*=316) clusters. Fewer participants were assigned to the SIRD (*n*=130) cluster and only a small number to the SIDD (*n*=18) cluster. The mean age was 54 (SD 10) years and 556 (65%) were male. The clinical characteristics of the GDS cohort according to the different type 2 diabetes subtypes are shown in Table 1.

**Table 1:** Characteristics of individuals with recent-onset type 2 diabetes in the GDS cohort, stratified by cluster allocation

Characteristic	SIDD (*n*=18)	SIRD (*n*=130)	MOD (*n*=316)	MARD (*n*=395)
Sex				
Male	61.1 (11) [38.6, 79.7]	66.2 (86) [57.7, 73.7]	53.8 (170) [48.3, 59.2]	73.2 (289) [68.6, 77.3]
Female	38.9 (7) [20.3, 61.4]	33.8 (44) [26.3, 42.3]	46.2 (146) [40.8, 51.7]	26.8 (106) [22.7, 31.4]
Age (years)	51.8 [47.6, 56.0]	57.1 [55.5, 58.7]	46.3 [45.2, 47.3]	58.3 [57.6, 59.0]
BMI (kg/m^2^)	29.5 [27.9, 31.2]	34.7 [33.8, 35.3]	35.5 [34.8, 36.2]	27.5 [27.2, 27.8]
HbA_1c_ (mmol/mol)	74.7 [70.1, 79.1]	44.1 [42.9, 45.4]	47.3 [46.2, 48.3]	45 [44.2, 45.7]
HbA_1c_ (%)	9.0 [8.6, 9.4]	6.2 [6.1, 6.3]	6.5 [6.4, 6.6]	6.3 [6.2, 6.3]
HOMA2-B	47 [38, 57]	187 [177, 196]	102 [98, 107]	89 [85, 92]
HOMA2-IR	2.8 [2.4, 3.3]	4.0 [3.7, 4.3]	2.8 [2.7, 2.9]	1.9 [1.9, 2]
SCORE2-Diabetes (%)	11.6 [8.8, 14.4]	10.4 [9.5, 11.3]	6.3 [5.8, 6.8]	10.3 [9.8, 10.7]

Data are % (count) [95% CI] or mean [95% CI]

### Classification uncertainty in the GDS cohort

**Fig. 3:**
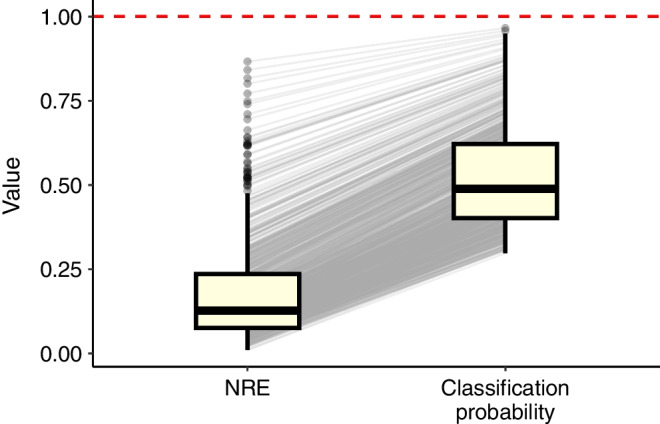
Boxplots of the NRE and the classification probability for the assigned cluster of individuals with recent-onset type 2 diabetes in the GDS cohort. The midline of the boxplot shows the median and the box shows the lower and upper quartiles. The whiskers extend to the minimum and maximum values within 1.5 times the IQR from the lower and upper quartiles. Grey lines connect each individual’s NRE value to their classification probability, illustrating the relationship between the two measures. Note that the NRE takes into account not only the classification probability for the assigned cluster but also the classification probabilities for the remaining three clusters. While both measures share the same maximum possible value of 1 (red dashed line), the minimum value for the NRE is 0, and for the classification probability, it is 0.25

The median NRE in the GSD cohort was 0.127 (95% CI 0.119, 0.135). The boxplot, shown in Fig. 3, is right-skewed, indicating that high NRE values were relatively uncommon. For comparison, the median classification probability for the assigned clusters of individuals was 48.8% (95% CI 47.3, 50.2) (Fig. 3). Additional graphical illustrations of the relationship between classification probability and NRE are available in ESM Figs 8, 9.

**Fig. 4:**
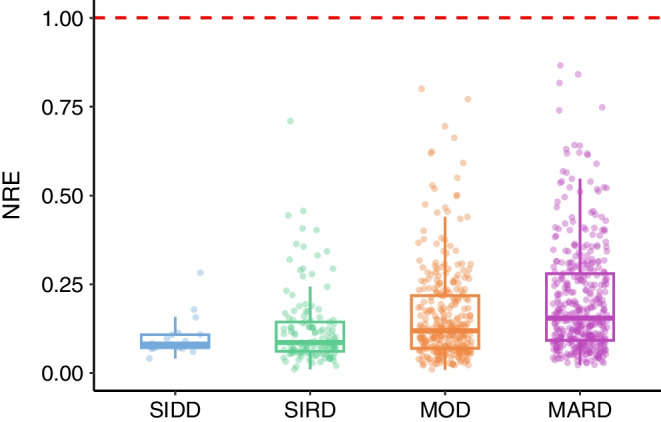
Boxplots of the NRE for individuals with recent-onset type 2 diabetes in the GDS cohort, stratified by the type 2 diabetes subtypes: SIDD (*n*=18), SIRD (*n*=130), MOD (*n*=316) and MARD (*n*=395). The midline of the boxplot shows the median and the box shows the lower and upper quartiles. The whiskers extend to the minimum and maximum values within 1.5 times the IQR from the lower and upper quartiles. The red dashed line indicates the maximum possible NRE

The boxplots of the NRE per cluster revealed considerable heterogeneity in classification uncertainty across the type 2 diabetes subtypes in the GDS cohort (Fig. 4). The median NRE was somewhat higher for the mild diabetes subtypes, with values of 0.155 (95% CI 0.142, 0.177) for the MARD cluster and 0.119 (95% CI 0.107, 0.131) for the MOD cluster. In contrast, the median NRE was lower for the severe diabetes subtypes, with values of 0.086 (95% CI 0.075, 0.108) for the SIRD cluster and 0.082 (95% CI 0.071, 0.109) for the SIDD cluster.

### Clinical features associated with low classification certainty

**Fig. 5:**
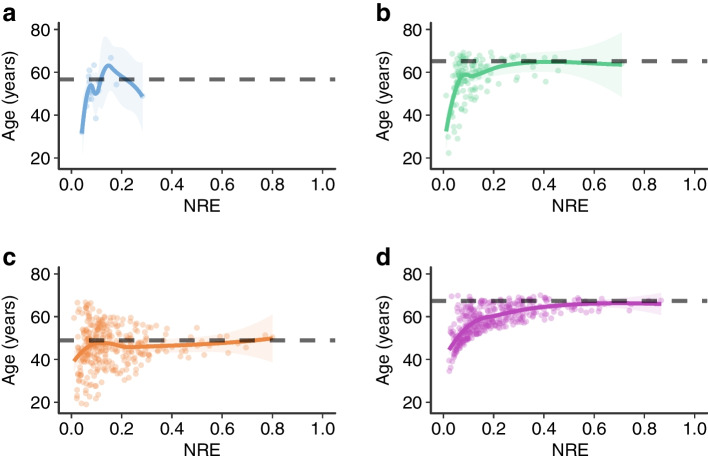
Association between the NRE and age across the different type 2 diabetes subtypes: (**a**) SIDD; (**b**) SIRD; (**c**) MOD; and (**d**) MARD. The black dashed line indicates the mean age of the respective subtype in the original ANDIS cohort. The solid line corresponds to a local polynomial regression fit separately for each subtype

For the different type 2 diabetes subtypes, the NRE showed distinct associations with the clinical features used for cluster assignment. For example, a lower NRE indicated a greater mismatch between an individual’s age and the typical age associated with its subtype (Fig. 5). Individuals in the MARD cluster with a low NRE were much younger than the typical age of 67 years reported in the ANDIS cohort [1]. For the MOD subtype, the mismatch occurred in both directions, as individuals with a low NRE were often either considerably younger or older than the typical MOD age of 49 years reported in the ANDIS cohort [1]. In both MARD and MOD, individuals with higher NRE values had ages that more closely aligned with the typical age of their assigned subtype.

Analogous patterns can be observed when examining the associations between the NRE and other clinical features (ESM Figs 10–13). For instance, in the SIRD cluster, individuals with a low NRE tended to have much lower insulin resistance than what is typical for this severe subtype of diabetes (ESM Fig. 11). For the SIDD subtype, no individuals in the GDS cohort exhibited the markedly elevated HbA_1c_ levels typically associated with this subtype, as reflected in their low NRE values (ESM Fig. 12).

### Accounting for classification uncertainty

**Fig. 6:**
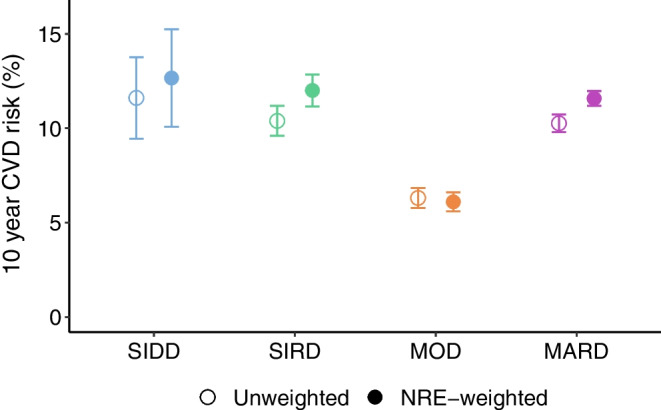
Predicted 10 year CVD risk (SCORE2-Diabetes) with 95% CIs for each subtype, based on a linear regression model with or without accounting for classification uncertainty via NRE-weighting. Predictions are derived from individuals with complete clinical data for SCORE2-Diabetes, with sample size as follows: SIDD (*n*=15); SIRD (*n*=111); MOD (*n*=256); and MARD (*n*=321)

The proportion of variation in the SCORE2-Diabetes explained by the subtypes (*R*^2^) increased from 17.4% (95% CI 12.8, 23.0) to 31.5% (95% CI 26.4, 37.1) after weighting individuals by classification certainty. The predicted SCORE2-Diabetes values for the different clusters, with and without NRE-weighting, are shown in Fig. 6. The differences in CVD risk between the type 2 diabetes subtypes were more pronounced in the NRE-weighted model compared with the unweighted model. The predicted 10 year CVD risk for the MARD subtype increased from 10.3% (95% CI 9.8, 10.7) in the unweighted model to 11.6% (95% CI 11.2, 12.0) in the NRE-weighted model. Similarly, for the SIRD subtype it increased from 10.4% (95% CI 9.6, 11.2) in the unweighted model to 12.0% (95% CI 11.2, 12.8) in the NRE-weighted model. Details of the unweighted and the NRE-weighted regression models are available in ESM Table 1.

## Discussion

In this study, we show that classification uncertainty can be assessed in three simple steps. First, individuals are assigned to subtypes using the established nearest centroid approach [1, 2]. Second, the resulting Euclidean distances are used to calculate classification probabilities for each of the four subtypes. Third, the NRE is computed based on all four probabilities, providing an overall measure of classification uncertainty for each individual. Since the Euclidean distances are calculated from sex-specific centroids [1], our measure of classification uncertainty inherently accounts for sex differences in diabetes subtypes.

In contrast to previous studies [15, 16], we propose and employ a continuous measure of classification uncertainty rather than relying on a discrete cut-off. This allows us to make explicit comparisons of uncertainty across the different subtypes. In the GDS cohort, the classification certainty is, on average, lower for the severe subtypes (SIDD and SIRD) than for the mild subtypes (MOD and MARD). This finding is plausible given that the GDS excludes individuals with HbA_1c_ levels greater than 75 mmol/mol (9%) at the baseline visit [14]. As a result, the SIDD subtype, which is characterised by higher HbA_1c_ levels (averaging 102 mmol/mol (11.5%) in the ANDIS cohort [1]), is less well represented in the GDS cohort. Additionally, GDS data were collected within 1 year of diagnosis and participants may have already initiated glucose-lowering therapy. This might have further contributed to lower HbA_1c_ levels and thus a lower classification certainty for individuals with SIDD. Similarly, the mean insulin resistance in the GDS cohort is lower than in the ANDIS cohort. Specifically, the mean HOMA-IR of the SIRD subtype in the GDS cohort is 4.0 (SD 1.5) compared with 5.5 (SD 2.7) in the ANDIS cohort [1]. This discrepancy contributes to greater distances to the SIRD centroid, resulting in lower classification certainties for the SIRD subtype in the GDS cohort.

More broadly, our findings from the GDS cohort illustrate that some degree of classification uncertainty is inevitable when individuals are assigned to discrete clusters. In other words, not every person matches equally well with the typical clinical profile of their assigned subtype. For example, individuals assigned to the SIRD subtype may still vary considerably in terms of their actual insulin resistance (see ESM Fig. 11). The low median NRE of 0.127 (95% CI 0.119, 0.135) in our approach results from the inverse distance-based probability estimation, which strongly penalises deviations from the ANDIS cluster prototypes (see ESM Methods). However, despite varying individual distances from cluster centroids, the type 2 diabetes subtypes group together individuals with similar pathophysiological characteristics, as has been validated with gold-standard measurements [2]. In terms of clinical outcomes, distinct clusters demonstrated associations with metabolic dysfunction-associated steatotic liver disease [2], nephropathy [2], neuropathy [2], cardiovascular risk [26] and erectile dysfunction [23], among others. In a recent study, findings from the GDS cohort also highlighted differences between the subtypes in patient-reported outcomes, including depression symptoms, diabetes-related distress and health-related quality of life [33].

In clinical practice, when considering subtype-specific risks for complications, it is important to simultaneously consider an individual’s classification uncertainty. If an individual has a low classification certainty, they might have a poor overlap with their assigned subtype in terms of their clinical features (see Fig. 5 and ESM Figs 10–13). For example, an overweight, elderly person might be assigned to the MOD subtype despite also sharing features of the MARD subtype. By recognising the lower classification certainty for the MOD subtype, clinicians can also focus on screening for complications that are more prevalent in the MARD subtype. Conversely, if an individual has a high classification certainty, it might be particularly important to screen them for specific complications associated with their assigned subtype.

In theory, this also extends to the notion of subtype-specific personalised treatment strategies, which have been proposed previously [34]. However, to date, there is no clear evidence to support the idea that the subtypes differ in their responses to specific treatments [3, 35]. For example, a recent clinical trial compared sodium–glucose cotransporter 2 inhibitors and glucagon-like peptide 1 receptor agonists specifically in individuals with SIRD and SIDD and found no significant interaction between treatment and subtype [35]. In such a subtype-specific trial, it would be highly relevant to report a measure of classification uncertainty, as this would provide information on how well the subtypes were represented in the trial cohort. In a sensitivity analysis, participants could be weighted by their classification certainty to account for heterogeneity in the observed clinical profiles of a subtype. In future subtype-specific trials, a minimum classification certainty could be used as an inclusion criterion to ensure participants align well with the subtypes of interest. Drawbacks to this approach include potential recruitment challenges and limited generalisability, particularly for individuals who do not fit well into these type 2 diabetes subtypes.

While it might be tempting to establish categories for the NRE, this is neither desirable [36] nor necessary when comparing clinical outcomes between subtypes. As demonstrated in our analysis of SCORE2-Diabetes, classification uncertainty can be accounted for by weighting individuals by their NRE. After weighting by classification certainty, the proportion of variation in SCORE2-Diabetes explained by the subtypes nearly doubles and the elevated risk in the MARD and SIRD subtypes becomes more pronounced. This is plausible, as individuals who do not match well with their subtype (e.g. younger individuals classified as MARD) contribute less to the analysis, while those with more representative clinical profiles contribute more. However, there is a trade-off inherent to this weighting procedure. On the one hand, NRE-weighting aligns the subtypes in the cohort more closely with the original ANDIS subtypes, which underlie the nearest centroid approach. On the other hand, the subtypes become less reflective of the specific cohort, such as the GDS cohort studied here. To illustrate this, histograms of each subtype’s clinical features in the GDS cohort before and after NRE-weighting are provided in ESM Figs 14–17. In this sense, the weighting procedure can be viewed as a form of standardisation towards the ANDIS prototypes. The decision to apply weights should depend on the research objective: whether the goal is to obtain cohort-specific results (e.g. tailored to the GDS cohort) or generalisable findings that align with the original subtype definitions.

Previous studies of classification uncertainty in the type 2 diabetes subtypes have produced mixed results. Tanabe et al [15] found that only for 14% of individuals in a Japanese cohort the cluster assignment was ‘undecidable’, which they defined as a classification probability of lower than 60%. When applying the same cut-off approach to the GDS cohort, we find that 72% of individuals would be categorised as ‘undecidable’. This reveals strong differences in the classification probabilities derived from the machine-learning model by Tanabe et al [15] and those derived from Euclidean distances. Instead, our results are closer to those of Wesolowska-Andersen et al [16], who reported that 65% of individuals in the European IMI-DIRECT cohort could not be clearly assigned to one of four subtypes.

These discrepancies in findings highlight a methodological challenge in quantifying classification uncertainty. In principle, there are different ways in which classification probabilities for the type 2 diabetes subtypes can be obtained. In our approach, we derive the classification probabilities from the inverse Euclidean distances to the cluster centroids [19]. However, alternative approaches could be considered, potentially yielding less-conservative measures of classification uncertainty. For example, methods based on exponential decay (softmax algorithm [37]) or Gaussian similarities (Radial Basis function kernel [37]) could be used. However, we find that our NRE measure performs well at identifying individuals who do not match well with their assigned subtype. Specifically, individuals who diverge in terms of key clinical features (e.g. low age for individuals with MARD) typically have a lower NRE. In contrast, individuals with a larger NRE align more closely with the previously reported cluster prototypes [1] (see Fig. 5).

Finally, it is important to note that the NRE is a relative measure of classification certainty and is not based on an absolute distance criterion. In particular, the classification probabilities of an individual depend on how far away the four cluster centroids are compared with each other. For example, two individuals may have the same Euclidean distance to the MARD centroid, yet their classification probability and NRE may differ depending on their distances to the other centroids (see ESM Fig. 18 for an example). When comparing classification uncertainty across cohorts with different distributions in the clustering variables, it might therefore be useful to also consider an absolute criterion such as the Euclidean distance to the assigned cluster.

The strength of our study lies in the proposal of a statistically robust measure of classification uncertainty. By accounting for uncertainty through the NRE, we mitigate some of the information loss inherent in hard clustering [10]. The NRE is easy to calculate and considers the degree of similarity to all four subtypes, offering an overall measure of classification uncertainty. It relies on readily available information that is directly obtained from the cluster assignment procedure. This makes it applicable in epidemiological studies and as a general diagnostic tool for assessing individual classification uncertainty. To facilitate its application, we plan to include the NRE in the recently published DDZ Diabetes-Cluster-Tool, a free online software for diabetes subtyping [18]. Currently, the tool already includes a visual display of an individual’s classification probabilities to enable an assessment of classification uncertainty.

While the NRE serves as a useful measure of classification uncertainty, it also has some limitations. First, although the NRE theoretically ranges from 0 to 1, it is a conservative measure and high values are difficult to achieve in practice. Therefore, it might be better suited for comparative evaluations (e.g. comparing classification uncertainty between clusters or individuals) and as a tool to account for classification uncertainty via weighting. By contrast, interpreting the absolute NRE value in isolation might be challenging and less informative. Second, the NRE does not offer detailed insights into how exactly an individual matches with their assigned subtype. For instance, two individuals with MARD may have the same NRE but different clinical profiles (ESM Fig. 19). To gain a more detailed insight, graphical tools such as spider charts provide a valuable complement. Third, although we demonstrate how to account for classification uncertainty using the SCORE2-Diabetes as an example, the 10 year CVD risk prediction relies solely on baseline features, without examining clinical outcomes during follow-up. Fourth, our analysis of the NRE is limited to a single cohort, which is not representative of the general diabetes population [14].

Future research should thus examine the NRE in other cohorts with more diverse clinical features. For example, while some classification uncertainty is inevitable with hard clustering, we expect higher certainty in the ANDIS cohort, where the clusters were originally derived [1]. More broadly, when new subclassifications are proposed (e.g. for impaired glucose tolerance [38] or type 1 diabetes [39]), NRE metrics should be reported to assess classification certainty and provide a benchmark for external validation cohorts. Finally, we acknowledge as a limitation that there was no patient or public involvement and engagement in the design and conduct of this study [40].

In conclusion, the NRE provides a means to quantify and compare individual classification uncertainty in type 2 diabetes subtypes. By raising awareness of classification uncertainty and providing a practical tool to assess it, we hope to enable a more careful application of diabetes subtypes in both research and clinical practice.

## Supplementary Information

Below is the link to the electronic supplementary material.

ESM (PDF 2050 KB)

## Data Availability

The dataset analysed in this study is not publicly accessible due to national data protection regulations and restrictions set by the ethics committee to safeguard the privacy of study participants. However, access can be requested through a specific project agreement with the principal investigator of the German Diabetes Study (GDS) (michael.roden@ddz.de). The study protocol and detailed methodology are published in the cohort profile [14] and are freely available.

## Abbreviations

ANDIS: All New Diabetes in Scania
GDS: German Diabetes Study
MARD: Mild age-related diabetes
MOD: Mild obesity-related diabetes
NRE: Normalised relative entropy
PMDI: Precision Medicine in Diabetes Initiative
SAID: Severe autoimmune diabetes
SIDD: Severe insulin-deficient diabetes
SIRD: Severe insulin-resistant diabetes

## Code availability statement

The R code of this study is available upon reasonable request from the corresponding author.

## Authors’ relationships and activities

KM received fees for consulting or lecturing from Eli Lilly, Novo Nordisk and Oviva, and performed investigator-initiated research with support from Sanofi-Aventis. MB received honoraria as a consultant and speaker from Amgen, AstraZeneca, Bayer, Boehringer Ingelheim, Daiichi Sankyo, Lilly, MSD, Novo Nordisk and Sanofi. MR received fees for consulting or lecturing from Astra Zeneca, Boehringer Ingelheim, Echosens, Eli Lilly, Madrigal, MSD, Novo Nordisk and Pfizer, and performed investigator-initiated research with support from Boehringer Ingelheim and Novo Nordisk. NS received fees for consulting or lecturing from Allergan, AstraZeneca, Boehringer Ingelheim, Gilead, Genkyotex, GSK, Intercept Pharma, Lilly, Merck Sharpe & Dohme, Novartis, Novo Nordisk, Pfizer and Sanofi, and received research support from AstraZeneca, Sanofi, DSM Nutritional Products and Roche Diagnostics. OPZ reports receiving lecture fees from Sanofi and Chiesi. RW reports receiving lecture fees from Novo Nordisk, Sanofi-Aventis, Boehringer Ingelheim and Eli Lilly, and has served on the advisory board for Akcea Therapeutics, Daiichi Sankyo, Sanofi-Aventis, Eli Lilly and NovoNordisk. SK reports receiving lecture fees from Berlin-Chemie, Lilly, Boehringer Ingelheim and JuZo-Akademie. SM received lecture fees from Eli Lilly and Novo Nordisk, and has participated on advisory boards for Novo Nordisk. SM’s husband is an employee at Novo Nordisk. The authors declare that there are no other relationships or activities that might bias, or be perceived to bias, their work.
