# Supplementary material for: Recognising, quantifying and accounting for classification uncertainty in type 2 diabetes subtypes
Source: Diabetologia. 2025 Jul 25;68(10):2139–50. doi: 10.1007/s00125-025-06486-4 (PMC12423224; doi:10.1007/s00125-025-06486-4)
Supplement: Supplementary file 1 — ESM (PDF 2050 KB) [file 125_2025_6486_MOESM1_ESM.pdf]

ESM Methods

Classification probabilities based on the nearest centroid approach

The Euclidean distances from the nearest centroid approach [1, 2] are computed based on the sex-specific cluster coordinates from the ANDIS cohort as reported by Ahlqvist et al [1]. For each individual we have a Euclidean distance (ED) to the center of the respective cluster.

$$ED_{SIDD}$$

$$ED_{SIRD}$$

$$ED_{MOD}$$

$$ED_{MARD}$$

The EDs measure the dissimilarity between an individual and the respective subtype. By inverting the EDs, we obtain a measure of similarity between an individual and the subtype with larger values indicating a smaller distance.

$$ED_{SIDD}^{Inv} = \frac{1}{ED_{SIDD}^2}$$

$$ED_{SIRD}^{Inv} = \frac{1}{ED_{SIRD}^2}$$

$$ED_{MOD}^{Inv} = \frac{1}{ED_{MOD}^2}$$

$$ED_{MARD}^{Inv} = \frac{1}{ED_{MARD}^2}$$

Since the NRE punishes low classification probabilities rather strictly, we proceed with the squared EDs, which lead to more distinct classification probabilities. To transform these similarity measures into classification probabilities, they are normalised [3] based on the sum of all inverse EDs

$$ED_{Total}^{INV} = ED_{SIDD}^{INV} + ED_{SIRD}^{INV} + ED_{MOD}^{INV} + ED_{MARD}^{INV}.$$

We then obtain the classification probabilities for each individual as

$$P(SIDD) = \frac{ED_{SIDD}^{Inv}}{ED_{Total}^{INV}}$$

$$P(SIRD) = \frac{ED_{SIRD}^{Inv}}{ED_{Total}^{INV}}$$

$$P(MOD) = \frac{ED_{MOD}^{Inv}}{ED_{Total}^{INV}}$$

$$P(MARD) = \frac{ED_{MARD}^{Inv}}{ED_{Total}^{INV}}.$$

Quantifying classification uncertainty using the normalised relative entropy

The relative entropy (also known as Kullback-Leibler divergence) [4] measures the distance between two probability distributions, p(x) and q(x), and is generally defined as

$$D(p||q) = \sum_{x \in X} p(x) \log \frac{p(x)}{q(x)}.$$

In our setting of interest, we want to compare the four classification probabilities of a given individual, p(x), to a reference scenario, q(x), with equal classification probabilities for each cluster. In such a setting with a uniform reference distribution, the relative entropy formula simplifies [4] to

$$D(p||q) = \log|X| + \sum_{x \in X} p(x) \log p(x),$$

where |X| is the number of clusters. Since there are four type 2 diabetes clusters, the formula becomes

$$D(p||q) = \log 4 + \sum_{x \in X} p(x) \log p(x).$$

If there is complete classification uncertainty (i.e. p(x) = 0.25 for all four clusters), the relative entropy reaches its minimum value of 0 [4]. In other words, the classification probabilities of the individual are identical to the reference scenario. Conversely, if there is no classification uncertainty (i.e. p(x) = 1 for one cluster and p(x) = 0 for the other three clusters), the relative entropy reaches its maximum value of log 4 [4]. In other words, the classification probabilities of the individual are as far away from the reference scenario as possible. Since the maximum possible relative entropy is known, we can normalise it [3] such that it falls between zero and one

$$\mathrm{NRE} = 1 + \frac{\sum_{x \in X} p(x) \log p(x)}{\log 4}.$$

Note that the second summand is always less than or equal to 0, ensuring that the NRE is always less than or equal to 1. The normalised relative entropy (NRE) is simple to calculate, as it only involves plugging in an individual's classification probabilities in the numerator (e.g. P(MOD) = 0.30) and summing it up for all four type 2 diabetes subtypes.

Normalised entropy-based measures are a versatile tool to quantify uncertainty and have previously been applied in network meta-analyses [5], psychiatric diagnoses [6] and ecological signal analysis [7]. For further mathematical details on the (normalised) relative entropy, see Cover and Thomas [4] and Kumar, Kumar and Kapur [3].

Relationship between the normalised relative entropy and classification probabilities

In general, the higher the classification probability for the assigned cluster, the higher an individual's NRE will be. However, there are two aspects worth noting. First, the association is non-linear and the NRE initially increases only slowly as a function of the probability of the assigned cluster (ESM Fig. 8). That is, low classification probabilities are punished rather strictly by the NRE and very high classification probabilities are required to achieve a high NRE.

Second, the NRE depends not only on the classification probability for the assigned cluster, but also on the distribution of the probabilities for the remaining clusters (ESM Fig. 9). Two individuals can have the same probability of being assigned to the MARD subtype, but different NREs due to their specific probabilities for the SIDD, SIRD and MOD subtypes. In particular, the more ambiguous the remaining probabilities are (i.e. similar probabilities for all three remaining clusters), the lower the NRE will be. In contrast, if there is a clear 2nd best candidate cluster (i.e. higher probability for one of the remaining clusters), the NRE will be higher. That way the NRE is able to distinguish between people at the border between two clusters (higher classification certainty) and people with a more ambiguous or less specific phenotype (lower classification certainty). This is useful, since individuals who do not fit neatly into any of the four subtypes will typically have more uniformly distributed classification probabilities and thus a lower NRE. Such an overall lack of fit with the Ahlqvist subtypes, however, might not be noticed if only one classification probability is considered. At the same time, this penalization of ambiguity further contributes to the NRE being a conservative measure of classification uncertainty. Note that this differentiation is more pronounced in settings with lower classification probabilities (ESM Fig. 9).

References

[1] Ahlqvist E, Storm P, Käräjämäki A, et al. (2018) Novel subgroups of adult-onset diabetes and their association with outcomes: a data-driven cluster analysis of six variables. Lancet Diabetes Endocrinol 6(5): 361-369. https://doi.org/10.1016/s2213-8587(18)30051-2

[2] Zaharia OP, Strassburger K, Strom A, et al. (2019) Risk of diabetes-associated diseases in subgroups of patients with recent-onset diabetes: a 5-year follow-up study. Lancet Diabetes Endocrinol 7(9): 684-694. https://doi.org/10.1016/S2213-8587(19)30187-1

[3] Kumar U, Kumar V, Kapur J (1986) Some normalized measures of directed divergence. Int J Gen Syst 13(1): 5-16. http://doi.org/10.1080/03081078608934950

[4] Cover TM, Thomas JA (2006) Entropy, relative entropy and mutual information. In: Elements of Information Theory. Wiley-Interscience, Hoboken, pp 13 - 55

[5] Wu Y-C, Shih M-C, Tu Y-K (2021) Using normalized entropy to measure uncertainty of rankings for network meta-analyses. Med Decis Making 41(6): 706-713. https://doi.org/10.1177/0272989X21999023

[6] Olbert CM, Gala GJ, Tupler LA (2014) Quantifying heterogeneity attributable to polythetic diagnostic criteria: theoretical framework and empirical application. J Abnorm Psychol 123(2): 452. https://doi.org/10.1037/a0036068

[7] Zaccarelli N, Li B-L, Petrosillo I, Zurlini G (2013) Order and disorder in ecological time-series: Introducing normalized spectral entropy. Ecol Indic 28: 22-30. http://doi.org/10.1016/j.ecolind.2011.07.008

ESM Tables

ESM Table 1 – Regression tables for predicting SCORE2-Diabetes based on the diabetes subtypes using either a standard linear regression model (unweighted) or an NRE-weighted linear regression model to account for classification uncertainty. In the regression models, MARD served as the reference group to which the other subtypes were compared.

	**Unweighted**				**NRE-weighted**			
Predictor	Estimate	SE	95% CI		Estimate	SE	95% CI	
			Lower limit	Upper limit			Lower limit	Upper limit
Intercept	10.3	0.2	9.8	10.7	11.6	0.2	11.2	12.0
SIDD	1.3	1.1	-0.9	3.6	1.1	1.3	-1.5	3.7
SIRD	0.1	0.5	-0.8	1.0	0.4	0.5	-0.5	1.4
MOD	-4.0	0.5	-4.7	-3.3	-5.5	0.3	-6.1	-4.8
MARD	-	-	-	-	-	-	-	-
	R^2 = 17.4% (95% CI: 12.8-23.0)				R^2 = 31.5% (95% CI: 26.4-37.1)			

ESM Figures

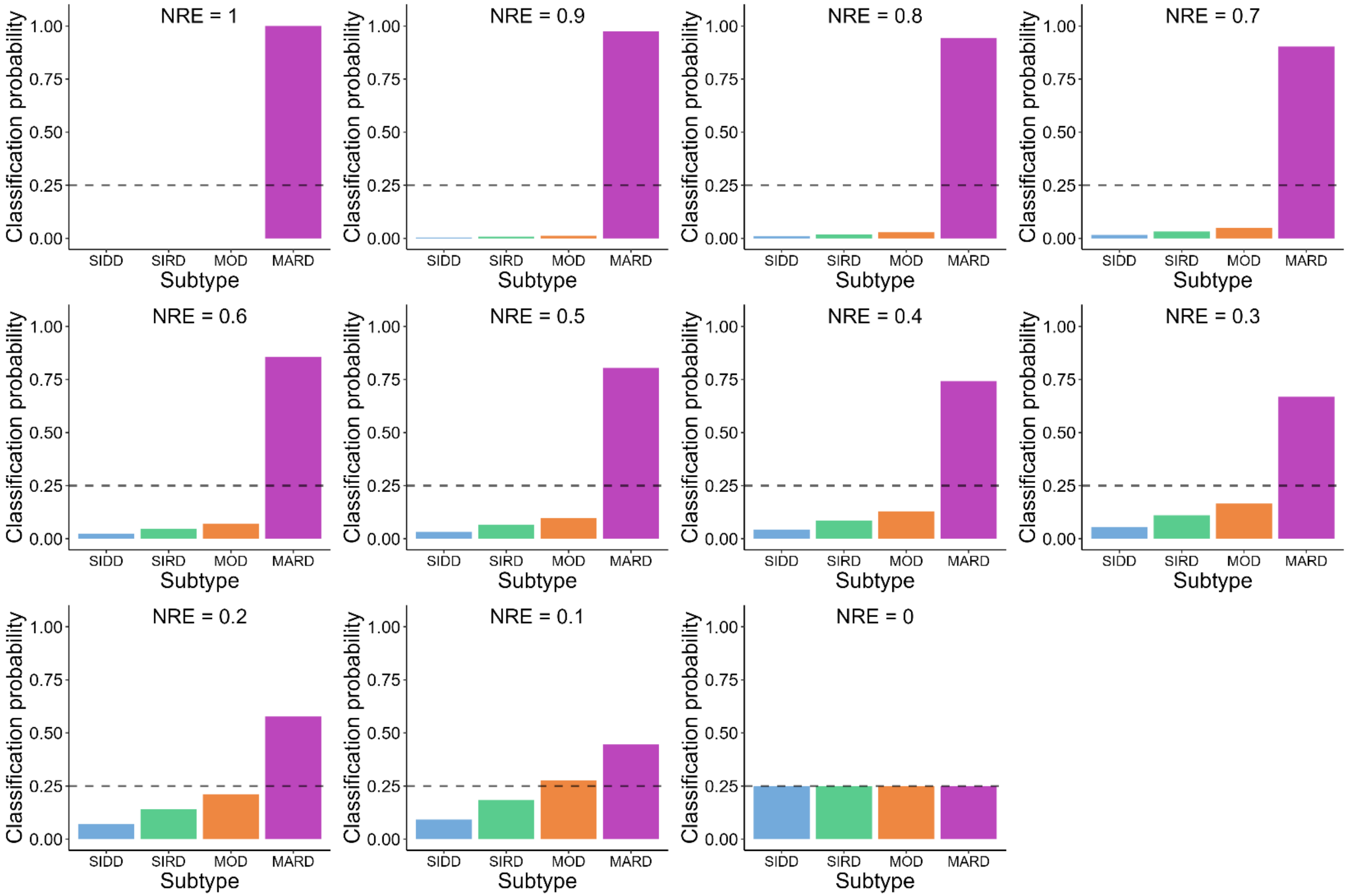

ESM Fig. 1 – Reference chart for NRE values ranging from one to zero. For this illustration, we assumed assignment to the MARD subtype and that the classification probabilities for the remaining clusters were linearly distributed (e.g. 5% - 10% - 15% - 70%).

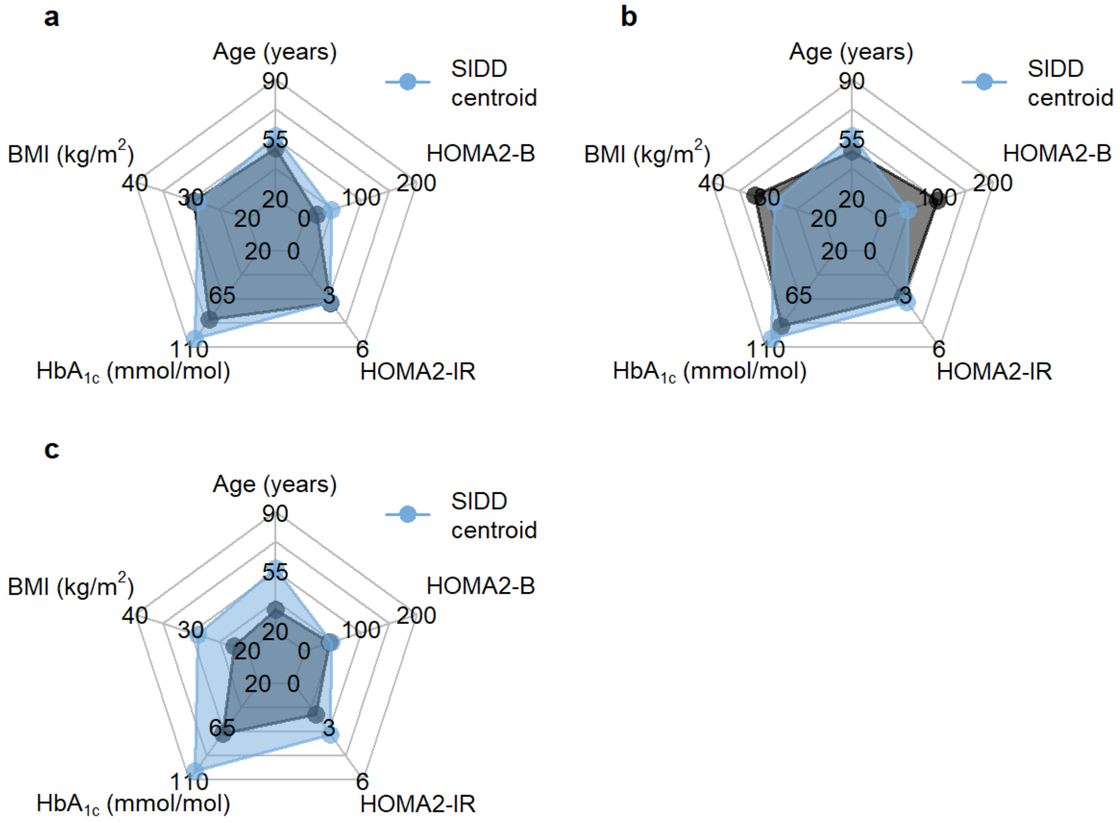

ESM Fig. 2 – Spider charts of three individuals from the GDS classified as SIDD. The charts display their clinical profiles (dark blue / black) in comparison with the typical SIDD profile from the nearest centroid algorithm (light blue).

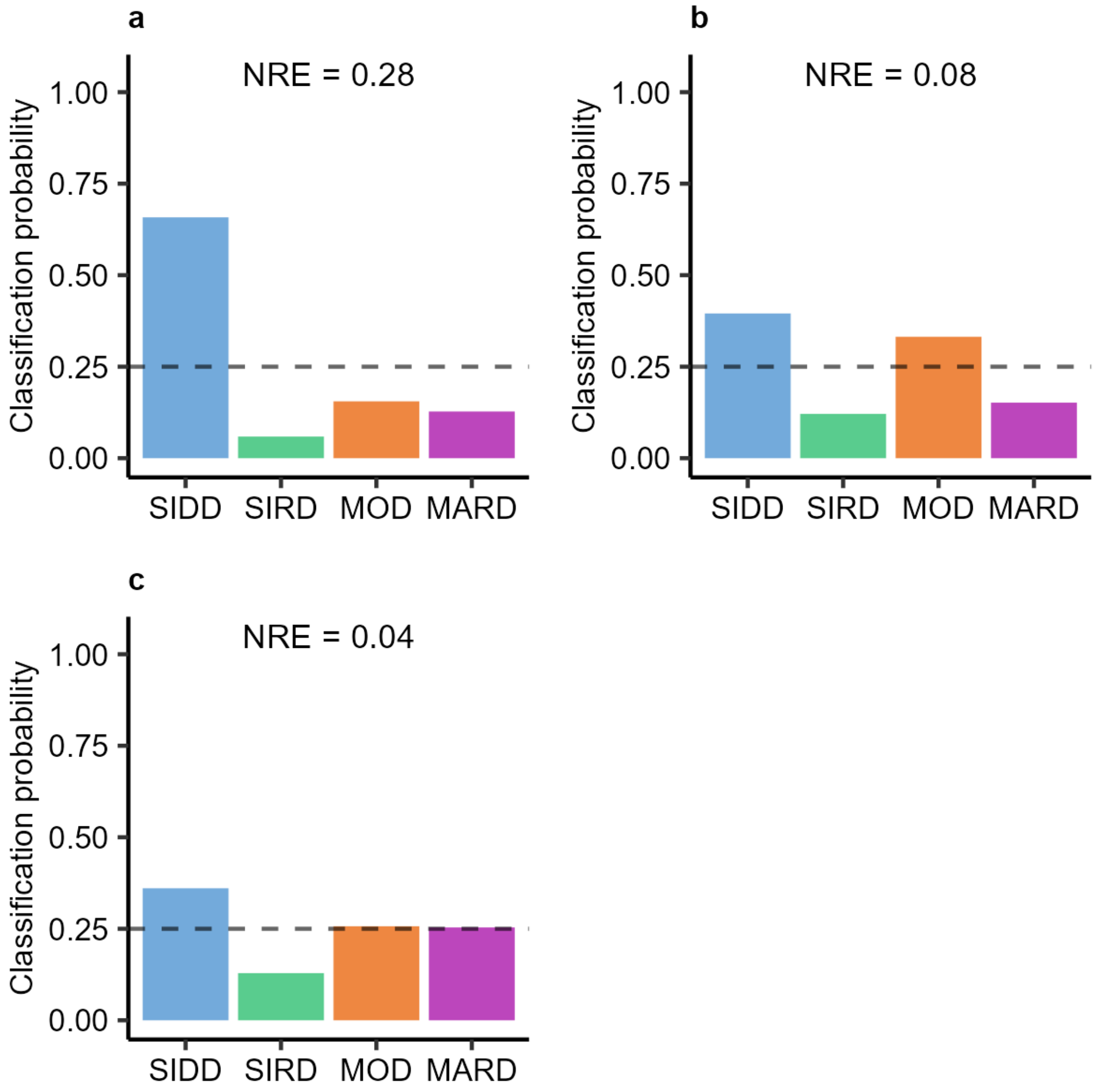

ESM Fig. 3 – Classification probabilities and NRE for three example individuals from the GDS assigned to the SIDD subtype by the nearest centroid algorithm. The NRE quantifies the classification uncertainty on a scale from 0 to 1, with higher values indicating greater certainty. The black dashed line indicates the classification probabilities in a reference setting with complete uncertainty regarding an individual's cluster assignment.

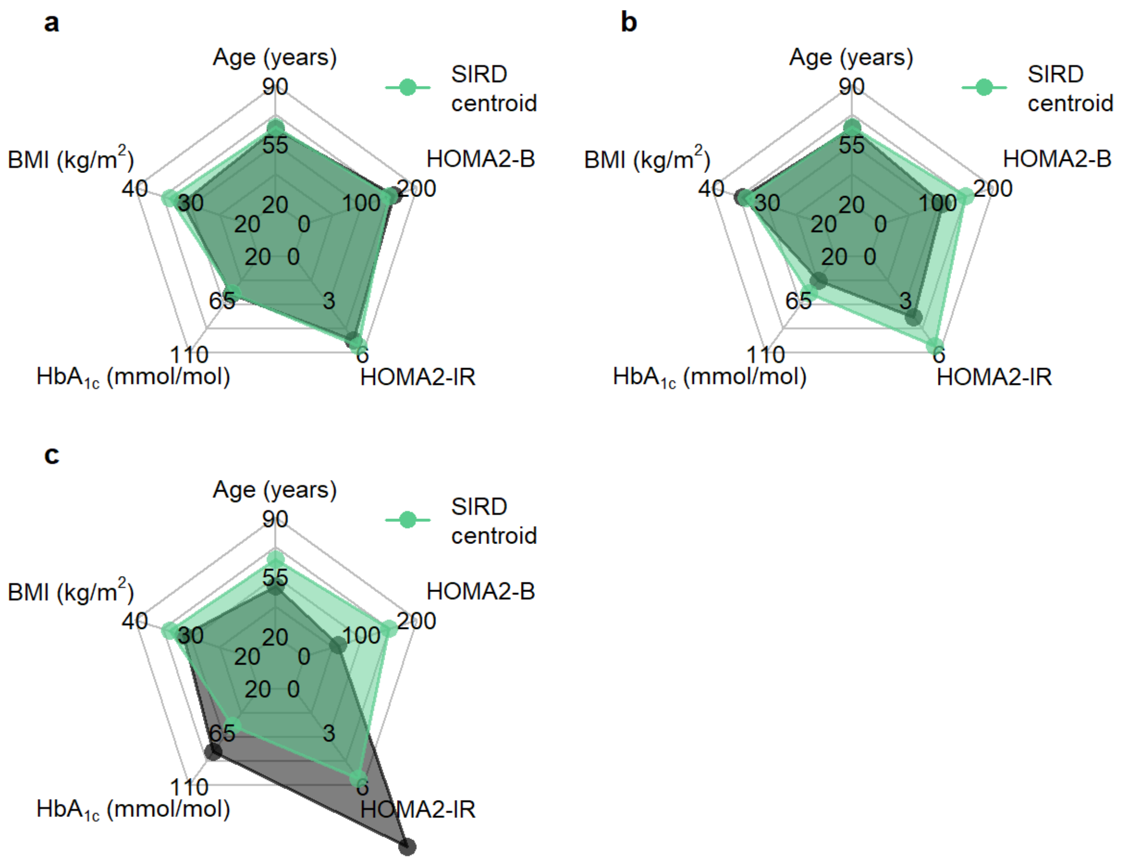

ESM Fig. 4 – Spider charts of three individuals from the GDS classified as SIRD. The charts display their clinical profiles (dark green / black) in comparison with the typical SIRD profile from the nearest centroid algorithm (light green).

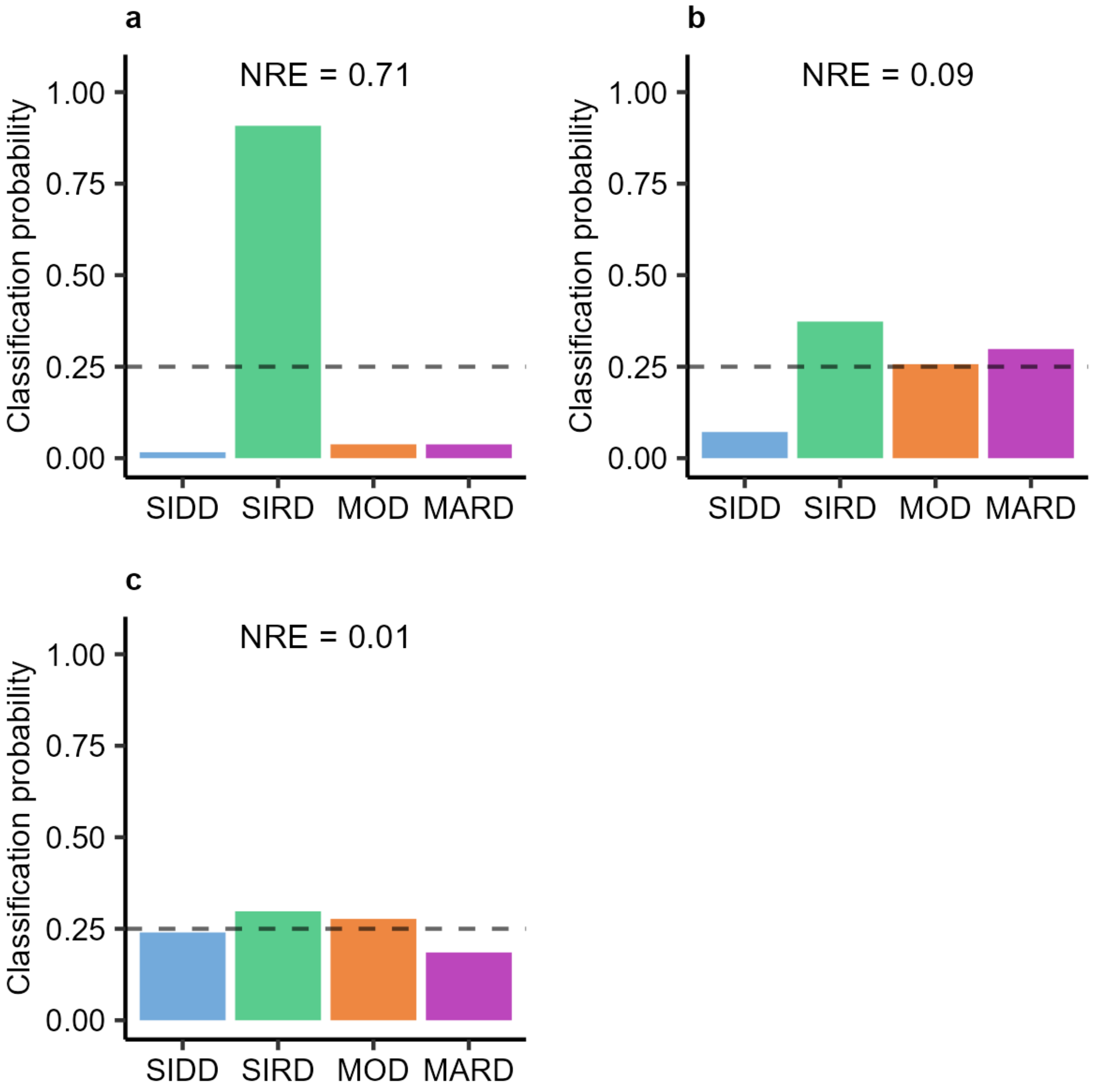

ESM Fig. 5 – Classification probabilities and NRE for three example individuals from the GDS assigned to the SIRD subtype by the nearest centroid algorithm. The NRE quantifies the classification uncertainty on a scale from 0 to 1, with higher values indicating greater certainty. The black dashed line indicates the classification probabilities in a reference setting with complete uncertainty regarding an individual's cluster assignment.

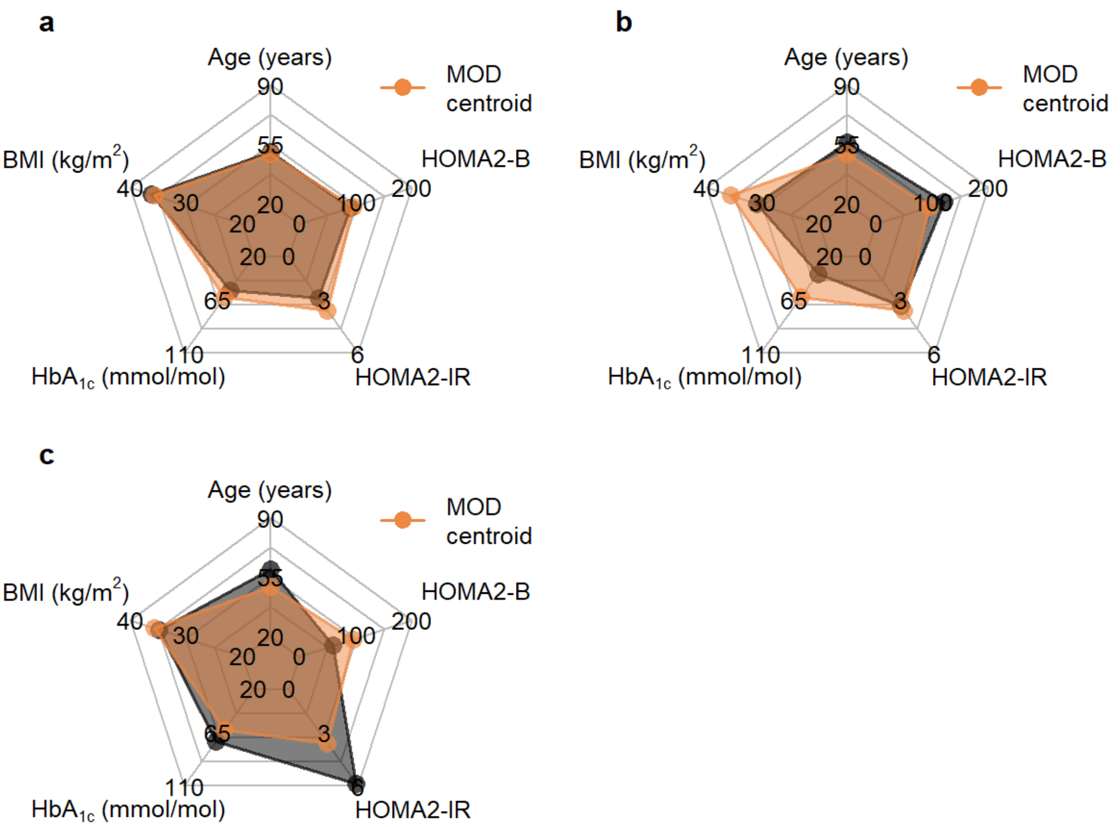

ESM Fig. 6 – Spider charts of three individuals from the GDS classified as MOD. The charts display their clinical profiles (dark orange / black) in comparison with the typical MOD profile from the nearest centroid algorithm (orange shape).

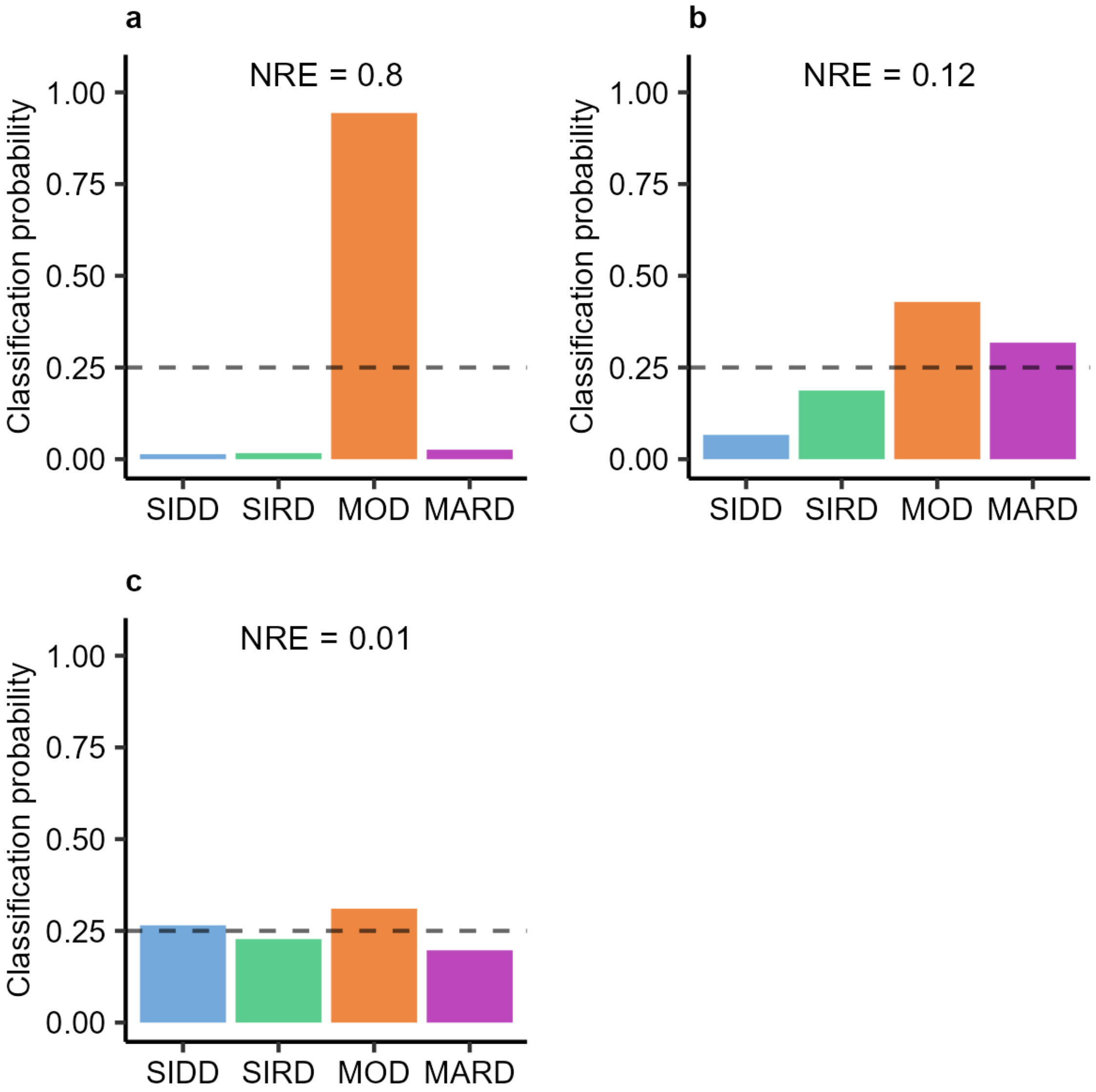

ESM Fig. 7 – Classification probabilities and NRE for three example individuals from the GDS assigned to the MOD subtype by the nearest centroid algorithm. The NRE quantifies the classification uncertainty on a scale from 0 to 1, with higher values indicating greater certainty. The black dashed line indicates the classification probabilities in a reference setting with complete uncertainty regarding an individual's cluster assignment.

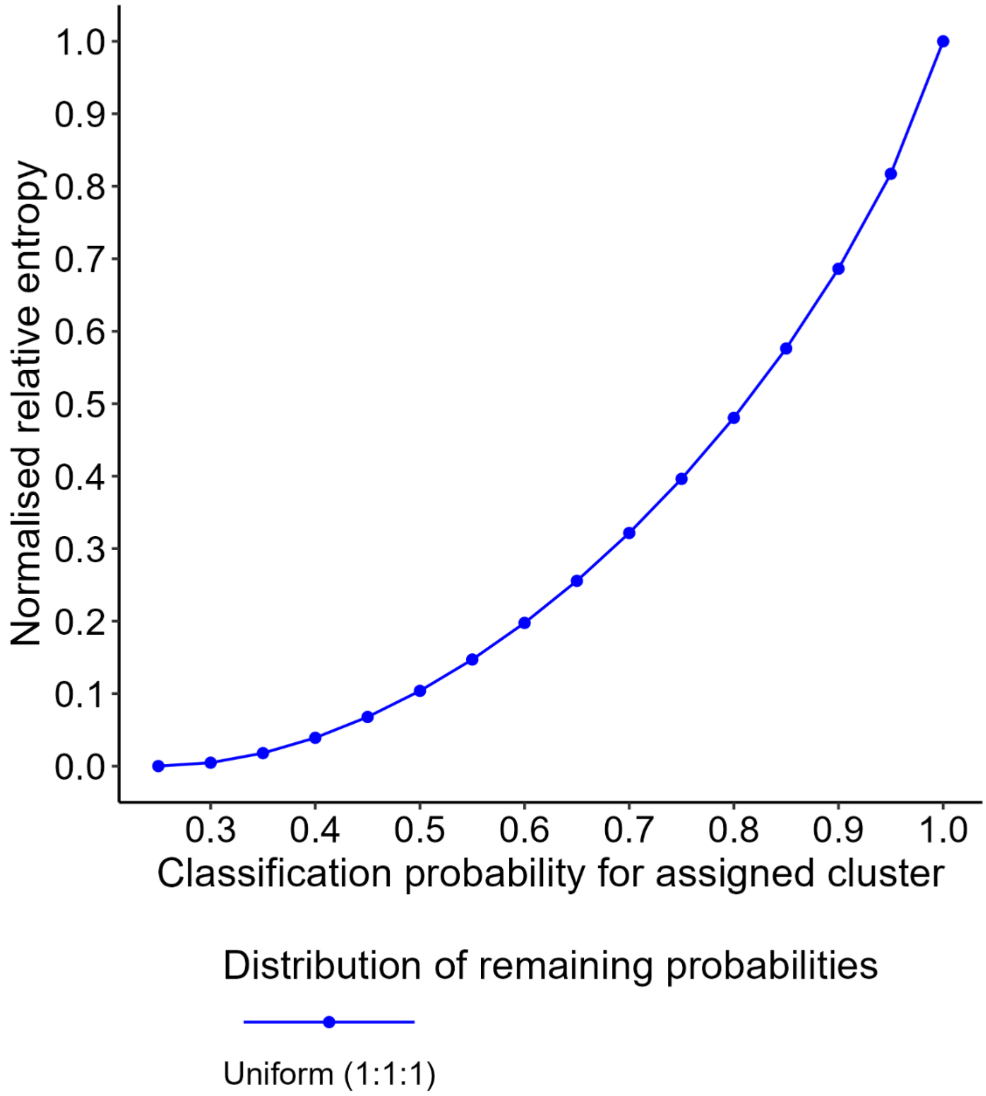

ESM Fig. 8 – Association between the classification probability for the assigned cluster and the NRE, assuming that the classification probabilities for the remaining clusters are uniformly distributed (e.g. 70% - 10% - 10% - 10%).

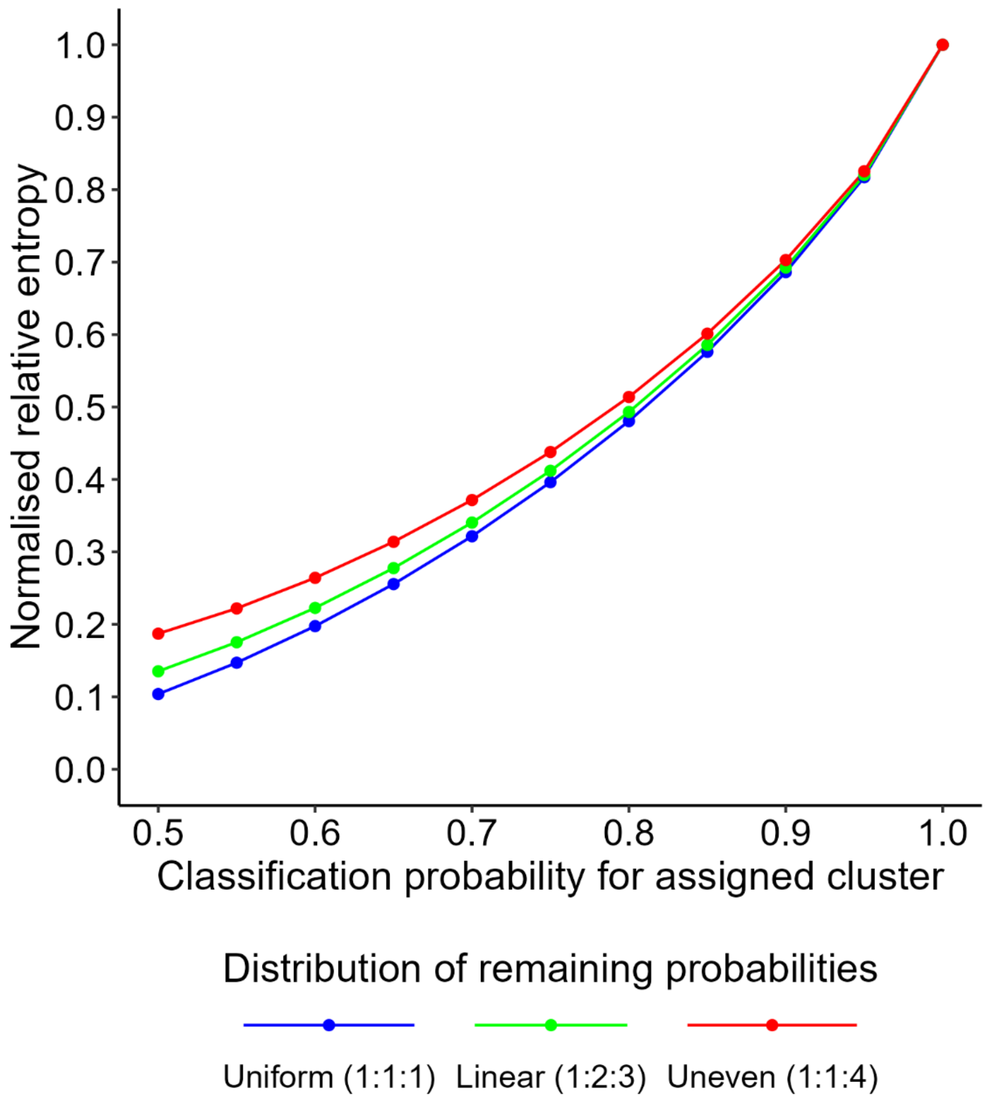

ESM Fig. 9 – Association between the classification probability for the assigned cluster and the NRE, assuming that the classification probabilities for the remaining clusters are either uniformly (e.g. 70% - 10% - 10% - 10%), linearly (e.g. 70% - 5% - 10% - 15%) or unevenly (e.g. 70% - 5% - 5% -20%) distributed.

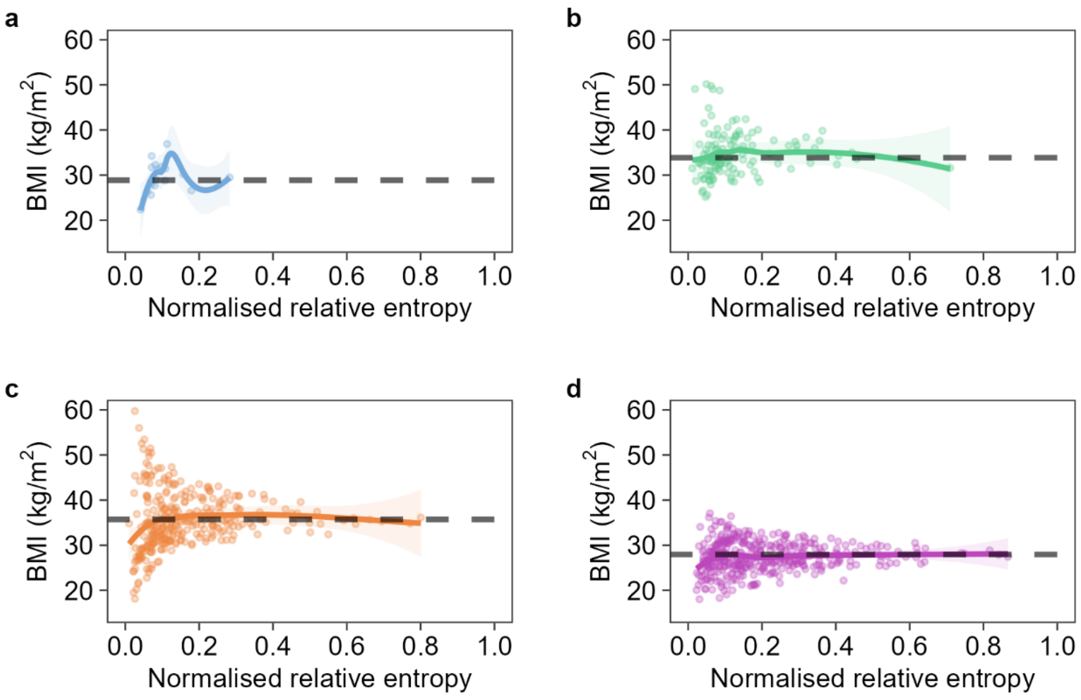

ESM Fig. 10 – Association between the NRE and BMI across the different type 2 diabetes subtypes. (**a**) SIDD; (**b**) SIRD; (**c**) MOD; and (**d**) MARD. The black dashed line indicates the mean BMI of the respective subtype in the original ANDIS cohort. The solid line corresponds to a local polynomial regression fit separately for each subtype.

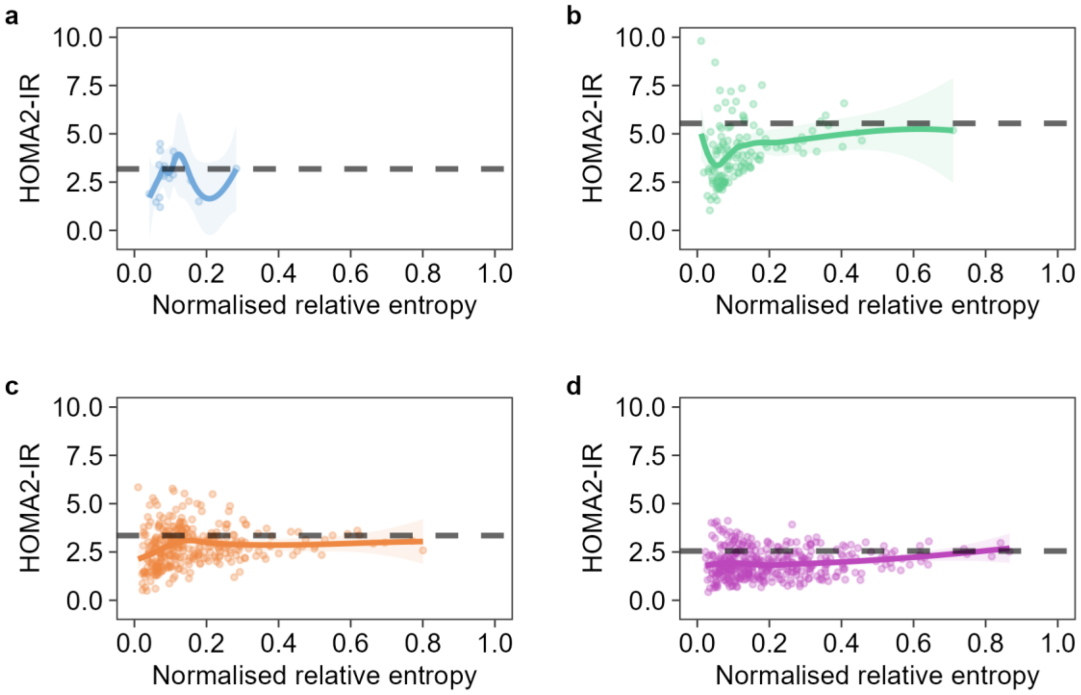

ESM Fig. 11 – Association between the NRE and HOMA-IR across the different type 2 diabetes subtypes. (**a**) SIDD; (**b**) SIRD; (**c**) MOD; and (**d**) MARD. The black dashed line indicates the mean HOMA-IR of the respective subtype in the original ANDIS cohort. The solid line corresponds to a local polynomial regression fit separately for each subtype.

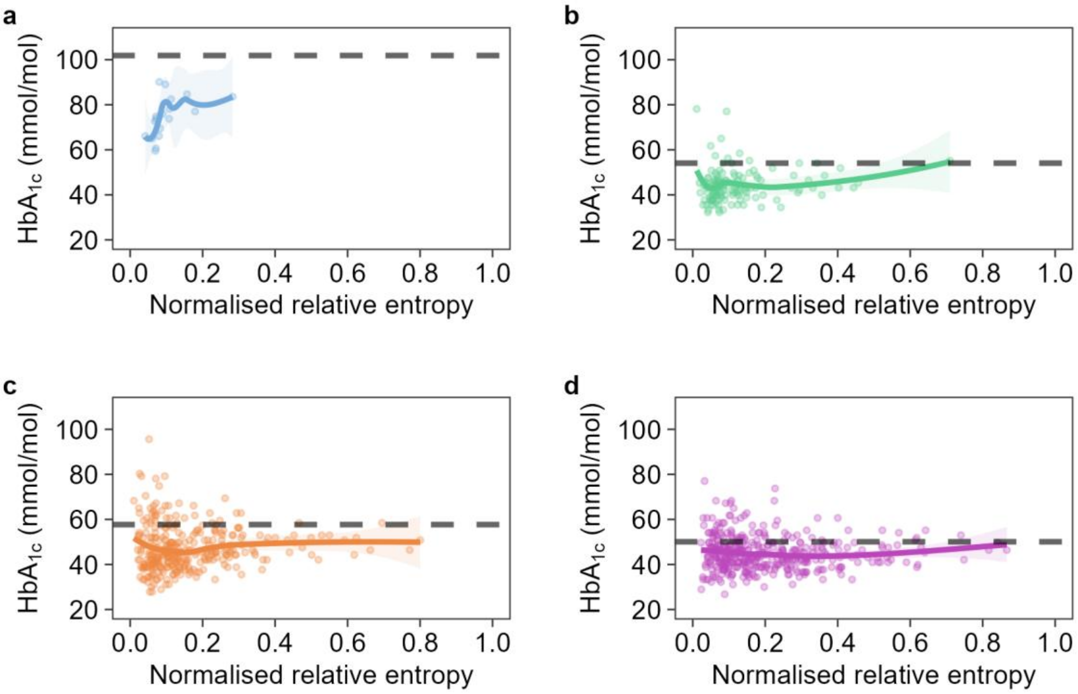

ESM Fig. 12 – Association between the NRE and HbA_{1c} across the different type 2 diabetes subtypes. (**a**) SIDD; (**b**) SIRD; (**c**) MOD; and (**d**) MARD. The black dashed line indicates the mean HbA_{1c} of the respective subtype in the original ANDIS cohort. The solid line corresponds to a local polynomial regression fit separately for each subtype.

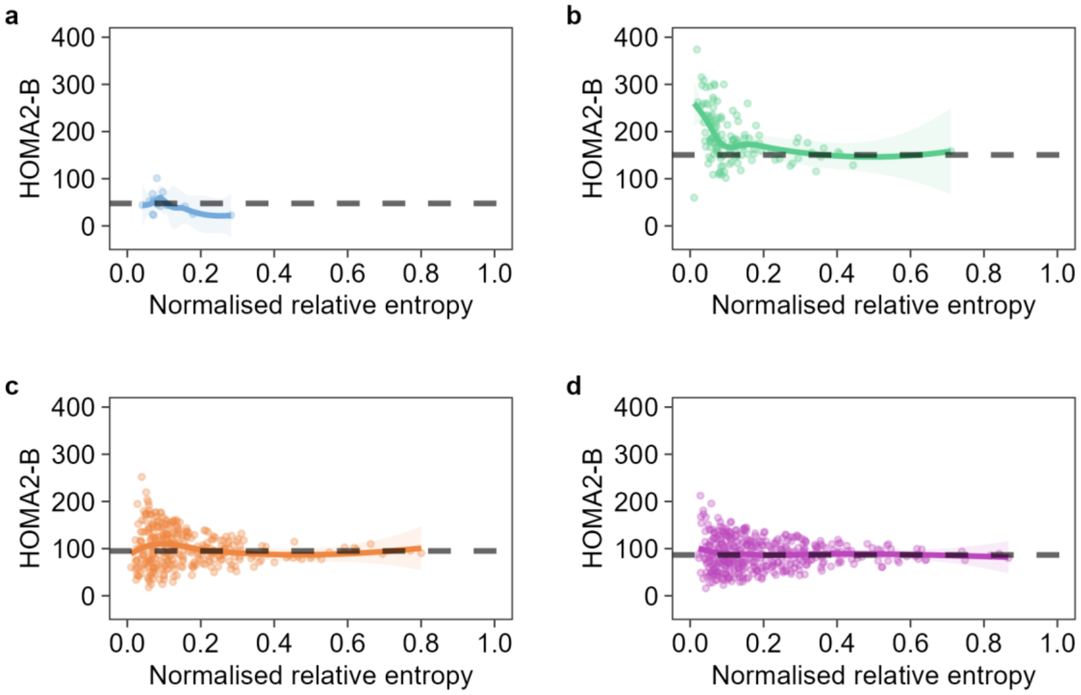

ESM Fig. 13 – Association between the NRE and HOMA-B across the different type 2 diabetes subtypes. (**a**) SIDD; (**b**) SIRD; (**c**) MOD; and (**d**) MARD. The black dashed line indicates the mean HOMA-B of the respective subtype in the original ANDIS cohort. The solid line corresponds to a local polynomial regression fit separately for each subtype.

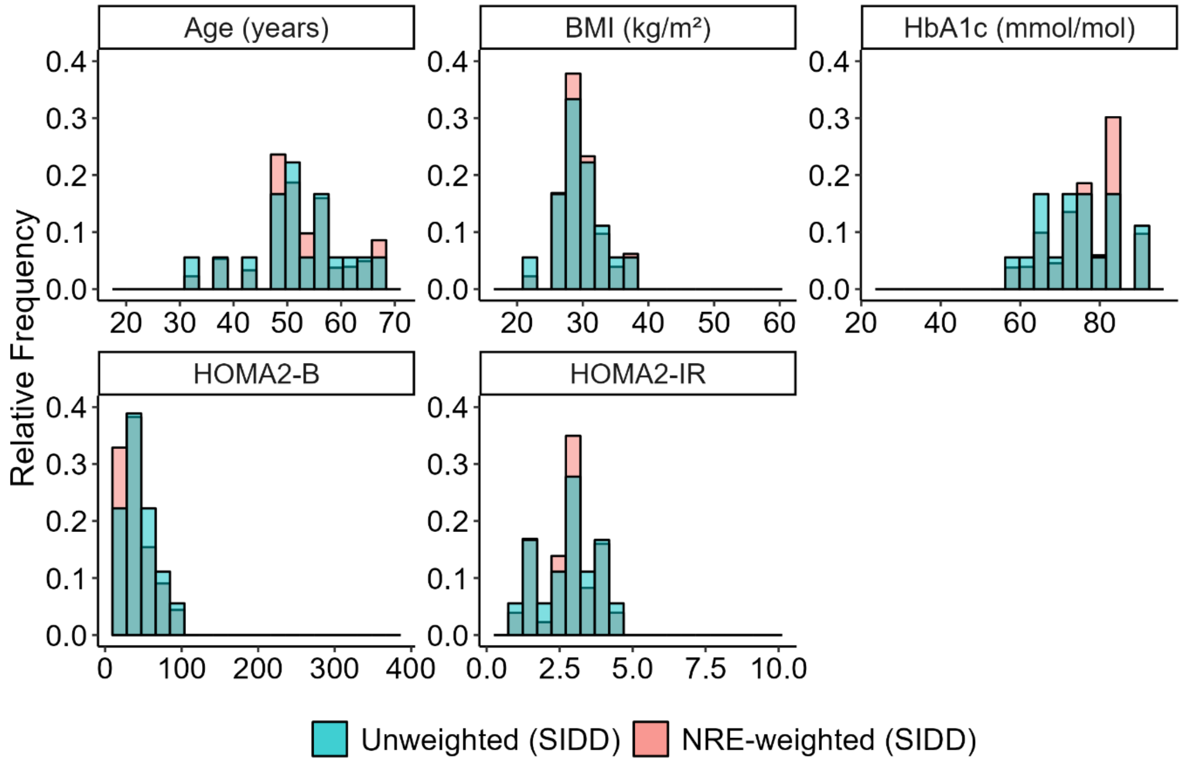

ESM Fig. 14 – Relative frequency histograms of the clinical features of the SIDD subtype in the GDS cohort before and after NRE-weighting. The histograms show where the unweighted and weighted distributions overlap and where they diverge.

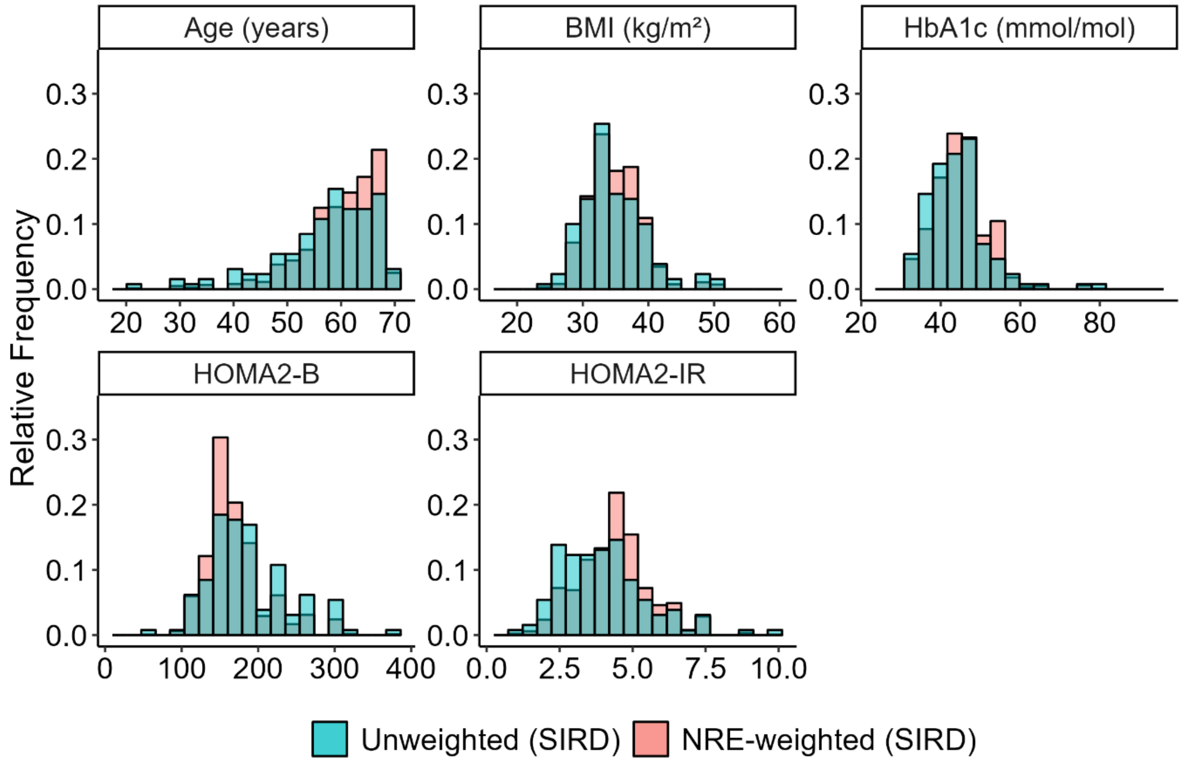

ESM Fig. 15 – Relative frequency histograms of the clinical features of the SIRD subtype in the GDS cohort before and after NRE-weighting. The histograms show where the unweighted and weighted distributions overlap and where they diverge.

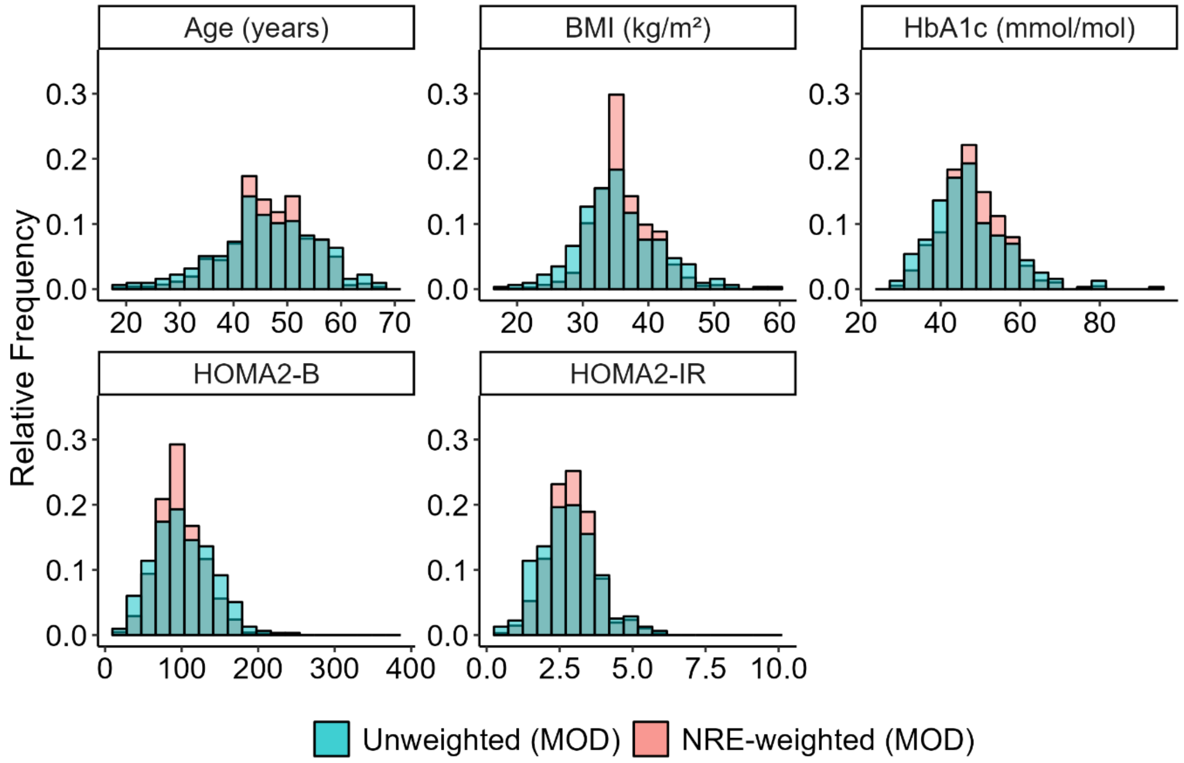

ESM Fig. 16 – Relative frequency histograms of the clinical features of the MOD subtype in the GDS cohort before and after NRE-weighting. The histograms show where the unweighted and weighted distributions overlap and where they diverge.

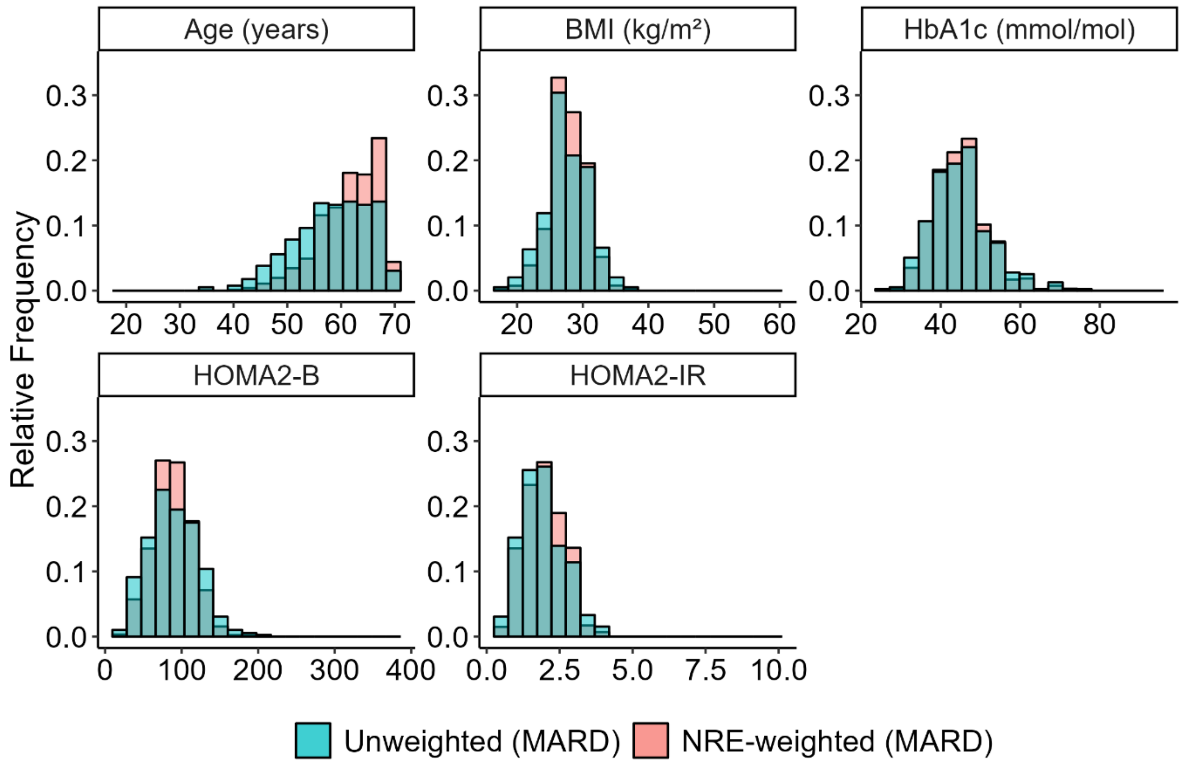

ESM Fig. 17 – Relative frequency histograms of the clinical features of the MARD subtype in the GDS cohort before and after NRE-weighting. The histograms show where the unweighted and weighted distributions overlap and where they diverge.

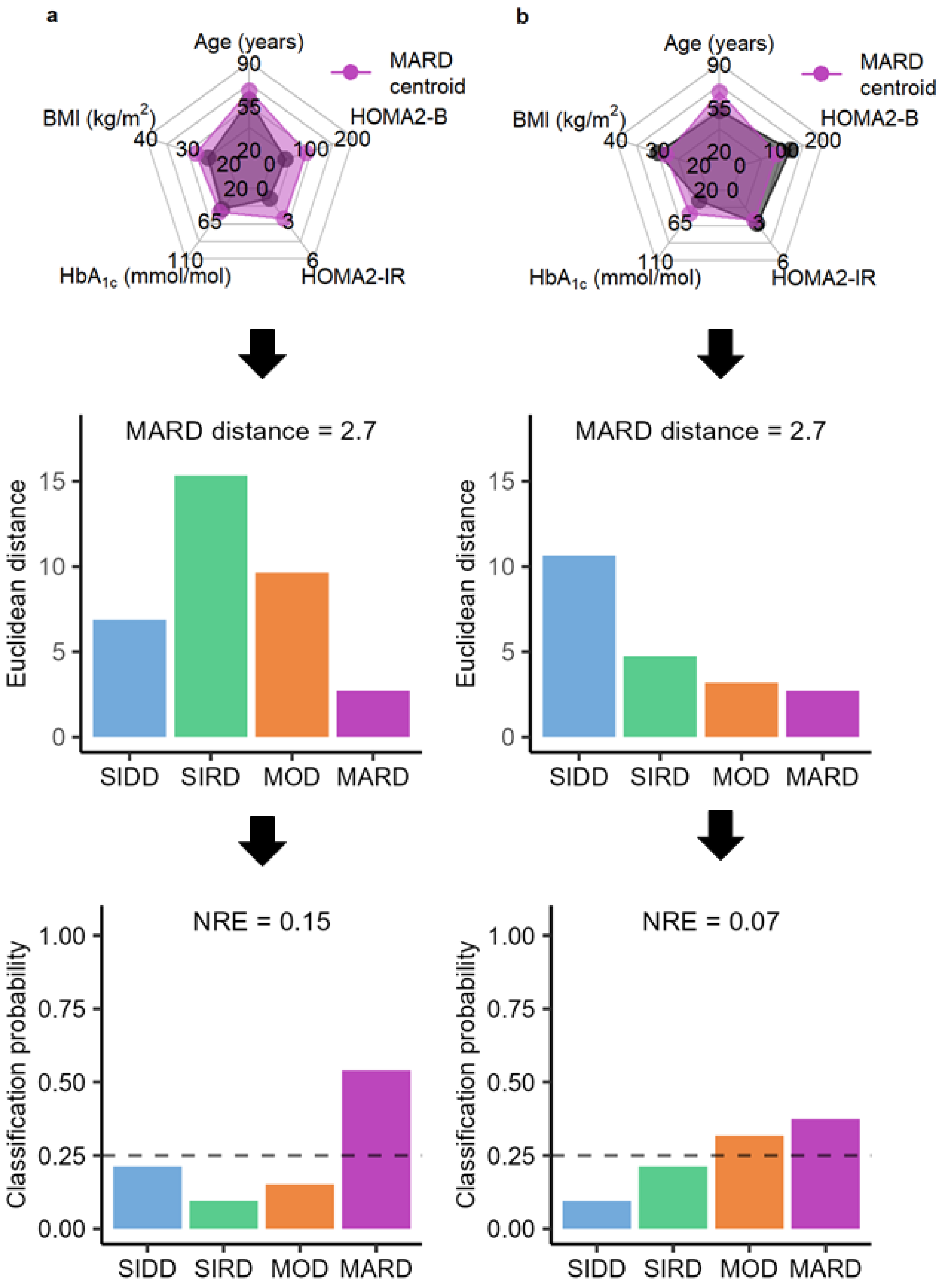

ESM Fig. 18 – Two MARD individuals from the GDS with the same Euclidean distance to their cluster centroid, but different classification probability and NRE. Person (**a**) is further away from the centroids of the remaining clusters, resulting in a higher classification probability for MARD (54%). Person (**b**) has a comparable Euclidean distance to the MOD and SIRD centroids, resulting in a lower classification probability for MARD (37%).

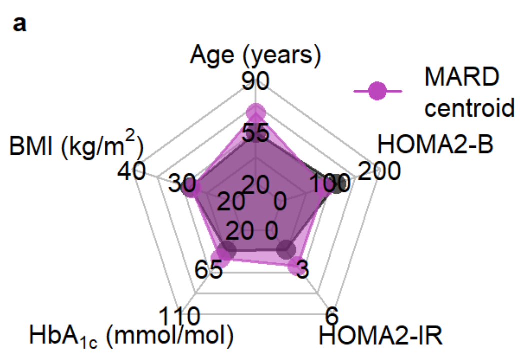

ESM Fig. 19 – Spider charts of two MARD individuals from the GDS with the same classification certainty (NRE = 0.14), but different clinical profiles. The charts display their clinical profiles (dark purple / black) in comparison with the typical MARD profile (light purple). The individual shown in graph a) is younger (54 vs 62 years), has a lower HbA_{1c} (41 vs 62 mmol/mol) and a higher insulin secretion (111 vs 30 HOMA-B) compared to the individual in graph b).
